# Mathematics Skills in Epilepsy: A Systematic Review and Meta-Analysis

**DOI:** 10.1007/s11065-023-09600-8

**Published:** 2023-07-25

**Authors:** Belinda J. Poole, Natalie L. Phillips, Brittany L. Killer, Camilla Gilmore, Suncica Lah

**Affiliations:** 1 School of Psychology, University of Sydney, Sydney, NSW 2006 Australia; 2 Centre for Mathematical Cognition, Loughborough University, Loughborough, UK

**Keywords:** Mathematics, Numeracy, Academics, Epilepsy

## Abstract

Mathematics incorporates a broad range of skills, which includes basic early numeracy skills, such as subitizing and basic counting to more advanced secondary skills including mathematics calculation and reasoning. The aim of this review was to undertake a detailed investigation of the severity and pattern of early numeracy and secondary mathematics skills in people with epilepsy. Searches were guided by the Preferred Reporting Items for Systematic Reviews and Meta-Analyses (PRISMA) statement. Twenty adult studies and 67 child studies were included in this review. Overall, meta-analyses revealed significant moderate impairments across all mathematics outcomes in both adults (g= -0.676), and children (g= -0.593) with epilepsy. Deficits were also observed for specific mathematics outcomes. For adults, impairments were found for mathematics reasoning (g= -0.736). However, two studies found that mathematics calculation was not significantly impaired, and an insufficient number of studies examined early numeracy skills in adults. In children with epilepsy, significant impairments were observed for each mathematics outcome: early numeracy (g= -0.383), calculation (g= -0.762), and reasoning (g= -0.572). The gravity of impairments also differed according to the site of seizure focus for children and adults, suggesting that mathematics outcomes were differentially vulnerable to the location of seizure focus.

## Introduction

Mathematics incorporates a broad range of skills, which begins with mastering early numeracy skills that later form the scaffolding to learning more advanced secondary mathematics skills (Geary, 2000). Early numeracy begins with basic quantitative skills, which typically emerge during infancy and preschool years. These skills include: subitizing (accurately determining a small number of items without counting), number comparison (rudimentary understanding of ordinality and magnitude of either symbolic items or non-symbolic items), counting, and simple arithmetic. During primary and secondary school, most children make further advances in secondary mathematics skills, such as: arithmetic computations (e.g. addition, subtraction, multiplication, and division of both single and multi-digit numbers) and using reasoning skills to solve mathematics word problems (Butterworth, 2005; Geary, 2000).

Pervasive and severe difficulties with mathematics can be the result of developmental dyscalculia or a mathematics learning disorder (MLD; Kaufmann et al., 2013; Kaufmann & von Aster, 2012), with two studies reporting a 6% prevalence rate of mathematics learning disorders in children (Fortes et al., 2016; Morsanyi et al., 2018). Some studies report that children with developmental dyscalculia, which only accounts for a subset of children with mathematics difficulties, often have a core deficit in early numeracy skills and struggle with the concept of numbers and quantities (Butterworth, 2005; Landerl et al., 2004). However, mathematics difficulties are heterogeneous and early numeracy skills may be intact with difficulties evident in secondary mathematics skills. According to the 5th text revision edition of the Diagnostic and Statistical Manual of Mental Disorders (DSM-5-TR; American Psychiatric Association [APA], 2022b) a diagnosis of Specific Learning Disorder with impairments in mathematics (i.e. MLD) can be provided with either evidence of impairment in early numeracy skills (e.g. poor number sense and difficulties memorizing arithmetic facts) or secondary skills (e.g. inaccurate or effortful calculation or problems with mathematics reasoning) that have persisted for six months or more despite intervention. Thus, children with MLD may have preserved abilities in these early numeracy skills yet struggle to make progress in mathematics during their school years.

Difficulties with mathematics can be explained by a range neurological, cognitive, and psychological reasons. Core deficits in early numeracy skills can stem from parietal lobe dysfunction, in particular the horizontal segment of the bilateral intraparietal sulcus, which has been found to be crucially important for processing early numeracy tasks (such as completing a number comparison task) and performing arithmetic calculation (Dehaene et al., 2003; Price et al., 2007). Studies have shown that activation of the left intraparietal sulcus increases with age in tasks that involve non-symbolic (e.g. dots) number processing (Ansari & Dhital, 2006) and with secondary mathematics skills, such as performing arithmetic (Rivera et al., 2005). As a result, those with developmental dyscalculia may have some disruption to this age-related maturation process in the frontoparietal regions of the brain (Ansari & Dhital, 2006).

However, not all mathematics difficulties stem from parietal dysfunction, but different subgroups of mathematical difficulties have been found to be related to a number of different underlying cognitive difficulties (Bartelet et al., 2014), including poor working memory capacity (Gathercole et al., 2016; McLean & Hitch, 1999; Menon, 2016). The involvement of working memory in mathematics has also been supported by two meta-analyses which examined imaging studies of brain regions that are involved in number processing and mathematics. These meta-analyses found that the cingulate gyrus was activated in calculation-based tasks, which is a region involved in working memory and attention (Arsalidou & Taylor, 2011; Arsalidou et al., 2018). Whilst these imaging studies revealed that parietal lobes are implicated in both number and calculation tasks, it was revealed that children performing mathematics tasks also activated diverse networks in the prefrontal cortex which include the working memory systems.

Working memory is a limited-capacity system that temporarily stores, processes, and manipulates information in mind. The traditional multicomponent model of working memory is made up of two temporary storage components: the phonological loop and a visuo-spatial sketchpad, which are coordinated by the central executive that processes and manipulates information in mind (Baddeley & Hitch, 1974; Baddeley, 1996, 2000) introduced a fourth component to the multicomponent working memory model: the episodic buffer, which binds episodic and semantic information into integrated chunks, and is an important interface with long term memory. A meta-analysis by Peng et al. (2016) found relationships between working memory and all types of mathematics skills, such as early numeracy, calculation, and problem solving in typically developing children. Furthermore, several reviews have reported that each working memory component differentially relates to performance on mathematics tasks, with the central executive crucially implicated across mathematics tasks (David, 2012; DeStefano & LeFevre, 2004; Friso-van den Bos et al., 2013; Raghubar et al., 2010). Taken together, each component of working memory is crucially implicated in performing mathematics tasks – such as holding numbers in mind in order to solve a calculation problem, it is also proposed to support various processes that underpin mathematics performance, such as retrieving arithmetic fact knowledge, computational procedural skills, and conceptual understanding of arithmetic principles (Cragg et al., 2017).

Another factor that may impede mathematics performance is mathematics anxiety, which refers to the fear and apprehension of mathematics that interfere with mathematics performance (Richardson & Suinn, 1972). Whilst there is limited evidence that test-specific anxiety adversely impacts academic test performance (e.g., Jerrim, 2022), mathematics anxiety is proposed to be distinguishable from general anxiety and test anxiety (Lukowski et al., 2016). Higher levels of mathematics anxiety has been associated with poorer mathematics achievement in both children and adults in a recent meta-analysis of 223 studies (Barroso et al., 2021). The causal relationship between mathematics performance and anxiety has been shown to be bi-directional (Foley et al., 2017). For instance, people may develop mathematics anxiety as a result of pre-existing difficulties with numbers and mathematics, which leads to continued avoidance of mathematics leading to greater problems with learning or performing mathematics in a range of situations (Luttenberger et al., 2018). In a small sample of young adults, high levels of mathematics anxiety resulted in lower working memory capacity, which impeded performance on mathematics tasks due to greater reaction times and errors in performing mental arithmetic (Ashcraft & Kirk, 2001). It was proposed that mathematics anxious individuals consumed their own working memory capacity with worry and related fears, such that they were unable to utilize those working memory resources to solve mathematics problems.

Whilst there is some evidence that there is a bi-directional relationship between poorer working memory and mathematics anxiety that impeded mathematics difficulties, not all children with poor working memory and mathematics difficulties present with higher levels of mathematics anxiety (Trickett et al., 2021). As a result, appropriately identifying and treating mathematics anxiety may assist in improving working memory resources to improve learning and mathematics performance if these are adversely impacted by mathematics anxiety.

### Mathematics Skills in Epilepsy

Epilepsy is one of the most common neurological diseases that involve both children and adults (Fiest et al., 2017). Epilepsy is characterized by recurrent unprovoked seizures (Fisher et al., 2014), which can be focal or generalized. Focal seizures emanate from a particular site (e.g. temporal or frontal lobe) within one hemisphere of the brain, whereas generalized seizures (which can be found in genetic generalized epilepsy [GGE]) rapidly propagate and engage diffuse networks in both hemispheres of the brain (Fisher et al., 2017). A review has shown that children with epilepsy are at risk for difficulties in academic learning and performance (Reilly et al., 2014), with a number of studies that found deficits in mathematics outcomes for both adults (Breier et al., 2000; Butterbaugh et al., 2004; Delazer et al., 2004) and children (Black & Hynd, 1995; Danguecan & Smith, 2017; Jackson et al., 2013; Rathouz et al., 2014; Seidenberg et al., 1986) with epilepsy. Yet few studies have investigated what mechanisms underpin those mathematics difficulties experienced in epilepsy. One hypothesis may be that people with parietal lobe epilepsy may experience disruption to processes that underlie number processing and mathematical skills. One study found that children with parietal lobe epilepsy were found to be below grade level in mathematics prior to epilepsy surgery (Sinclair et al., 2005), suggesting that parietal lobe epilepsy may lead to mathematics difficulties in those cases. However, parietal lobe dysfunction may not be a parsimonious explanation given that parietal lobe epilepsy is rare, accounting for only 6% of epilepsy cases in one study (Rasmussen, 1987). Furthermore, mathematics difficulties has also been documented in epilepsies that do not emanate from the parietal lobes, such as temporal lobe epilepsy (TLE; e.g. Miranda & Smith, 2001), frontal lobe epilepsy (FLE; e.g. Braakman et al., 2013), and in GGE (e.g. Jackson et al., 2013; Rathouz et al., 2014). This suggests that parietal lobe dysfunction for mathematics difficulties may not apply to those cases.

There are alternative explanations to mathematical difficulties to explore in epilepsy. For instance, people with epilepsy can experience a range of cognitive difficulties, which can be differentially impacted by the type of epilepsy and the associated structural abnormalities (e.g. tumor) or other epilepsy clinical features, such as age of seizure disorder onset, duration of epilepsy, seizure frequency, and side effects from anti-seizure medications (ASMs) (Badawy et al., 2012; Vingerhoets, 2006). For instance, working memory is critical for the development of mathematics skills and is known to be impaired across focal epilepsies and GGE. A recent meta-analysis found that children with epilepsy had global impairments in working memory – that is, impairments are found in all three components of working memory: the phonological loop, visuo-spatial sketchpad and central executive (Poole et al., 2021). However, the gravity and pattern of working memory deficits differed according to the site and side of seizure focus. The phonological loop was found to be the most disrupted, irrespective of the site and side of seizure focus, with the greatest magnitude of impairment found for children with FLE and bilateral TLE compared to typically developing children. The visuo-spatial sketchpad was found to be impaired in FLE, bilateral TLE, and in GGE. Similarly, the central executive was impaired in TLE, FLE and GGE. In children with unilateral TLE, the meta-analysis showed no evidence of deficits in the visuo-spatial sketchpad or central executive, albeit this lack of deficit may be due to the small number of studies included. An insufficient number of studies examined these components in extra-TLE/FLE (i.e. parietal and occipital lobe epilepsies). Furthermore, a younger age of onset was found to be related to reduced working memory capacity in both temporary storage components: phonological loop and visuo-spatial sketchpad. Longer duration of epilepsy was related to poorer visuo-spatial sketchpad capacity.

Given that mathematics skills relies, in part, on working memory - impairments of working memory place people with epilepsy at risk difficulties with early numeracy and secondary mathematics skills. Indeed, two studies have found a relationship between poor working memory capacity and difficulties with mathematics in children with epilepsy (Danguecan & Smith, 2017; Fastenau et al., 2004), however, both studies used a mixed sample of epilepsy cases, and examined secondary mathematics skills with a composite score of working memory. Thus, the role of each component of working memory in relation to different mathematics outcomes, including early numeracy skills, remains unknown. This is important, because working memory components are differentially impaired according to site and side of seizure focus in pediatric epilepsy (Poole et al., 2021), with each working memory component implicated in different mathematics tasks (Peng et al., 2016; Raghubar et al., 2010). For instance, children with unilateral TLE with intact central executive function, may perform better in tasks of mathematics problem solving than children with FLE or GGE whose central executive is impaired (Poole et al., 2021). Greater nuance in understanding the impact of working memory and mathematics difficulties in different types of epilepsy can inform detailed assessment and targeted interventions for those difficulties.

With respect to anxiety, a recent meta-analysis revealed that children and adolescents with epilepsy experience higher rates of clinical anxiety than the general population (Scott et al., 2020). Whilst mathematics anxiety is purported to be separate to general clinical and test anxiety, there are some overlap in proposed processes (e.g. worry) and does correlate with mathematics performance (Lukowski et al., 2016). Given the higher rates of clinical anxiety present in epilepsy, it is plausible that there may be higher rates of mathematics anxiety in children with epilepsy due to those overlapping processes. It is important to investigate in people with epilepsy, particularly as mathematics anxiety is also known to impact working memory and mathematics performance in the general population (Ashcraft & Kirk, 2001).

The extent and gravity of mathematics problems in epilepsy is currently unclear as most studies have focused on one mathematics outcome. It remains unknown if early numeracy or all secondary mathematics skills are also impaired in epilepsy and if the pattern of mathematics skills impairment differs for focal and generalized epilepsies, and if epilepsy variables disrupt mathematics outcomes. It is important to investigate which components of mathematics are impacted, so that appropriate supports can be provided. Furthermore, the relationship between early numeracy and secondary mathematics skills with other factors, such as: working memory, other cognitive skills, and mathematics anxiety are unclear. These findings could assist clinicians and educators in early identification and intervention of poor mathematics outcomes at school, and advocate for greater support for children and adults with epilepsy with poor mathematics skills.

The primary aim of this review is to evaluate and quantify the gravity of deficits in mathematics skills, such as early numeracy and secondary mathematics skills, in adults and children with epilepsy and determine whether those skills are differentially impacted according to site of seizure focus. The secondary aim is to determine whether early numeracy and mathematics skills are related to demographic and other epilepsy related factors, such as: age at testing, age of onset of seizure disorder, duration of epilepsy, seizure frequency, ASMs, and surgical status. The final aim of this review will examine whether early numeracy and mathematics outcomes in epilepsy were related to cognitive skills (e.g., working memory) or mathematics anxiety.

## Method

### Protocol Registration

This systematic review and meta-analysis protocol was registered on the Prospective Register of Systematic Reviews (PROSPERO; registration number CRD42019123294). The searches, data-extraction, and reporting of results were guided by the Preferred Reporting Items for Systematic Reviews and Meta-Analyses (PRISMA) 2020 statement (Page et al., 2021).

### Search Strategies

Databases including PsychInfo, SCOPUS and Web of Science were searched via OvidSP to identify eligible studies. The last search was conducted on the 8th of July 2022. The following Medical Subject Headings (MESH) and keyword search terms were used: [(exp. Epilepsy or epilep*.mp) AND (exp. Math*.mp OR Arithmetic*.mp OR Algebra*.mp OR Numeracy*.mp OR Numbers.mp (Numerals) OR Word Problem.mp* OR Mathematical.mp Achievement.mp OR exp. Mathematics (Concepts) OR exp. Mathematics Anxiety OR exp. Mathematical Ability OR exp. Academic Achievement)]. All MESH terms were exploded to include narrower MESH terms. Studies were limited to those that used human participants and were published in peer-reviewed journals in the English language. Results from SCOPUS were limited to “MEDI” OR “PSYCH” OR “MULT” subject areas. The reference lists of eligible papers and relevant reviews were searched to identify additional studies for inclusion.

### Study Selection Criteria

Studies were included if they: (i) reported original empirical research; (ii) included people with a diagnosis of epilepsy (including epilepsy syndromes) irrespective of pre-existing conditions and comorbidities; (iii) used an objective assessment of specific early numeracy skills (e.g. subitizing and number comparisons), or secondary mathematics skills (e.g. whole number calculations, arithmetic, fractions, geometry, algebra and mathematics problem solving) using a standardized psychometric assessment, or school based academic assessment, or using an experimental task; (iv) included a neurologically healthy control group, or reported scores that could be compared to normative data from a standardized test battery; and (v) were published in peer-reviewed journals in the English language.

Studies were excluded if they: (i) were review papers or single case studies; (ii) were dissertations, abstracts, or conference presentations; (iii) included composite scores of academic attainment (e.g. reading and mathematics outcomes), but did not publish or provide data (via e-mail request) for specific mathematics outcomes, or did not publish or provide values in order to calculate an effect size; (iv) had participant samples that overlapped with previous published studies; and (v) used a subjective measure of mathematics outcomes, such as parent or teacher questionnaires.

### Selection

Articles were imported, reviewed and data was extracted using Covidence Systematic Review Software (Veritas Health Innovation, 2022). The titles and abstracts were assessed against inclusion criteria by one reviewer (BP). A second reviewer (NP) screened a random 20% selection of titles and abstracts to ensure inter-rater reliability using Cohen’s kappa coefficient. Agreement was high at 91.1%, and inter-rater reliability was substantial at 0.8 (Landis & Koch, 1977). The full-text article was obtained if studies met inclusion criteria or if there was insufficient information in the title and abstract to determine whether the study met inclusion orexclusion criteria. Studies that clearly did not meet inclusion or exclusion criteria according to one or both raters were not reviewed at the full-text stage. Two reviewers (BP and NP) assessed all full text articles against inclusion criteria and the final decision was determined by consensus.

### Data Items and Summary Measures

The following data were extracted from each eligible article: (i) age at testing, (ii) sample size of epilepsy and control groups; and (iii) relevant epilepsy variables e.g. epilepsy diagnosis or site of seizure focus, age of seizure onset, duration of seizures, seizure frequency, and treatment (e.g. medication or surgery). Outcome data were also extracted from eligible articles: (i) mathematics test scores used to calculate an effect size (e.g. means and standard deviations, standard scores, or t-values and p-values); (ii) cognitive skills (e.g. working memory); and (iv) anxiety measures (i.e. general anxiety or mathematics-specific anxiety).

**Fig. 1:**
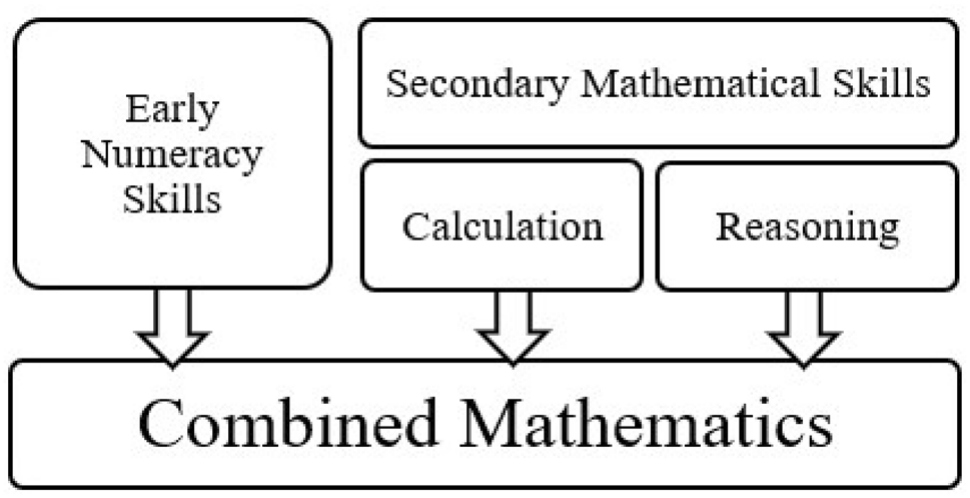
Classification of mathematical outcomes included as subgroups in meta-analysis

Each mathematics task was classified according to the criteria provided in the DSM-5 (American Psychiatric Association [APA], 2022a) for Specific Learning Disorder with impairment in mathematics. The DSM-5 provides four criteria for impairments that can be used for diagnosis of a mathematics-specific learning disorder: (i) number sense; (ii) memorization of arithmetic facts; (iii) accurate or fluent calculation; and (iv) accurate mathematics reasoning. In this review, tasks were classified into either (i) Early numeracy – which includes both number sense and memorization of arithmetic facts; and secondary mathematics skills, which included (i) calculation (combined accuracy and fluency tasks) of abstract tasks (i.e., presented only with digits); and (ii) mathematics reasoning, which included word problems presented in both verbal and written forms. Finally, all the aforementioned outcomes were combined into an overall effect size (iii) combined mathematics (see Fig. 1). The combined mathematics score also included tasks that evaluated multiple outcomes (e.g., composite mathematics scores made up of calculation and mathematics reasoning), and other mathematics outcomes that could not be clearly assigned to a subgroup (e.g. school grades). Classification of tasks were completed by one author (BP) and verified by a second author (SL). Classification was achieved by reviewing the manuals of standardized tasks, or the method section for experimental tasks, and determining the main outcome evaluated. Any disagreement in classification was discussed until a consensus was reached.

### Quality Analysis

The Newcastle-Ottawa Scale (NOS; Wells et al., 2000) was used to determine the quality of each study. The Cochrane collaboration has recommended the NOS to assess the quality and risk of bias in non-randomized and observational studies (Higgins & Green, 2011). One reviewer (BP) rated all studies and a second reviewer (BK) reviewed 20% of papers to ensure inter-rater reliability using Cohen’s kappa coefficient. Agreement was high at 92.93%, and inter-rater reliability was substantial at 0.86 (Landis & Koch, 1977). The NOS utilizes a star system to assess study quality, giving a score between 0 and 9 stars. For studies that did not utilize a comparison group, a numeric score (e.g., 2/2) was provided instead of a star to assess study quality. A higher number of stars or higher numeric score suggests better quality and less risk of bias. Risk of bias is assessed across three domains: selection, comparability, and exposure.

For *Selection* a maximum of four stars was awarded for studies with a control group, or a maximum of 2 out of 2 awarded for studies that did not have a control group and compared scores to normative data. One star was allocated for each of the following: (i) diagnosis of epilepsy is determined using more than one source or record (e.g. clinical assessment, or neuroimaging findings); (ii) recruited participants with epilepsy from consecutive referrals or referrals that are representative of the sample; (iii) controls were selected from the same community (or not applicable for studies involving normative data or no comparison group; N/A); and (iv) controls were defined as being neurologically healthy, or with no history of epilepsy or seizures (or N/A for studies that used normative data).

For *Comparability* a maximum of two stars was allocated for each of the following: (i) study controlled for age at testing; and (ii) study controlled for any other factor in the analyses. For studies that had no comparison group or used normative data, they received N/A for this category.

For *Outcome*, a maximum of three stars was awarded for studies with a comparison group, or a maximum score of one out of one awarded for studies that did not have a control group and instead compared scores to normative data. One star was allocated for each of the following criteria for: (i) reported an outcome that measured at least one aspect of mathematics or numeracy skill, using a standardized assessment or an experimental assessment that was described in enough detail to be replicated; (ii) utilized the same assessment measure for both cases and controls (or N/A); and (iii) reported the same non-response rate for both cases and controls (or N/A).

### Data Analysis

Comprehensive Meta-Analysis (CMA) software, version 3.3.070 was used to analyze data (Borenstein et al., 2014). Hedges’ *g* was used to calculate the standardized mean difference in mathematics or numeracy test scores between epilepsy and control groups. Hedges’ *g* was chosen as it corrects for biases in small samples that can lead to an overestimation of standardized mean differences (Borenstein, 2009). Effect sizes were interpreted as small (0.2), moderate (0.5) and large (0.8), consistent with interpreting Cohen’s *d* (Cohen, 2013; Perdices, 2018). Negative effect sizes revealed worse performance in the epilepsy group compared to controls. The meta-analysis used a random effects model and a two-tailed significance level was set at *p* < .05. Heterogeneity was evaluated using the Q statistic, Tau (T), Tau squared (T^2^), and the *I*^2^ statistic. Prediction intervals were used to report variation in effect size across studies (Borenstein et al., 2017).

The primary analyses examined each mathematics and numeracy outcome for the pooled epilepsy group, separately for children (mean age < 18 years), and adults (mean age ≥ 18 years). Subgroup analyses were also conducted, with mathematics and numeracy outcomes evaluated as a function of epilepsy type: (i) focal: temporal lobe epilepsy [TLE], frontal lobe epilepsy [FLE]; and focal epilepsies outside the frontal-temporal lobes [Extra-FLE/TLE]; (ii) generalized: genetic generalized epilepsy [GGE], previously known as idiopathic generalized epilepsy [IGE].

If studies reported more than one measure of a numeracy or mathematics skill (e.g. multiple measures of calculation outcomes), an average effect size was calculated. To ensure that analyses were not over-inflated, for those studies that had more than one epilepsy subgroup, but only had one control group or used normative data, the number of participants in the control group or normative data sample was divided evenly across each epilepsy group. Further, for studies that had data replicated or samples overlapped across different publications, only the main study (chosen as either the most complete in terms of outcomes or largest sample size) was used in the analyses.

Meta-regressions were conducted for continuous moderator variables, including age at testing, age of onset, and duration of seizure disorder. Due to the small number of studies, a systematic review of the remaining moderator variables, such as seizure frequency, ASMs, surgical outcomes, and other cognitive skills and mathematics anxiety was completed.

Publication bias was assessed by examining funnel plots and using Egger’s regression test (Egger et al., 1997) with significant results (p < .05) indicating asymmetry is present with the studies. A further sensitivity analysis was completed to examine whether the combined mathematics outcome across all epilepsy participants were influenced by the risk of selection bias from the NOS criteria. It is not clear what degree of bias is introduced if different criteria were not met under each category, thus using the full scale can be problematic (Lundh & Gøtzsche, 2008). In order to differentiate between high- and low-quality studies, the items from the *Selection* criteria were used. To ensure that the meta-analysis is generalizable to epilepsy populations, a well-defined epilepsy and control sample that were carefully recruited with minimal selection bias is important. The NOS does not provide a threshold score that differentiates between good and poor quality studies (Wells et al., 2000). *Selection* contains 4 items for studies that used a control group for comparison, or only 2 items for studies that used normative data. Thus, studies with the highest number of endorsed items (i.e. a rating of 2/2 or 3/4 or 4/4) were considered High *Selection* quality and the remaining studies (i.e. a rating of 1/2 or 1/4 or 2/4) were considered Low *Selection* quality.

## Results

### Study Selection

**Fig. 2:**
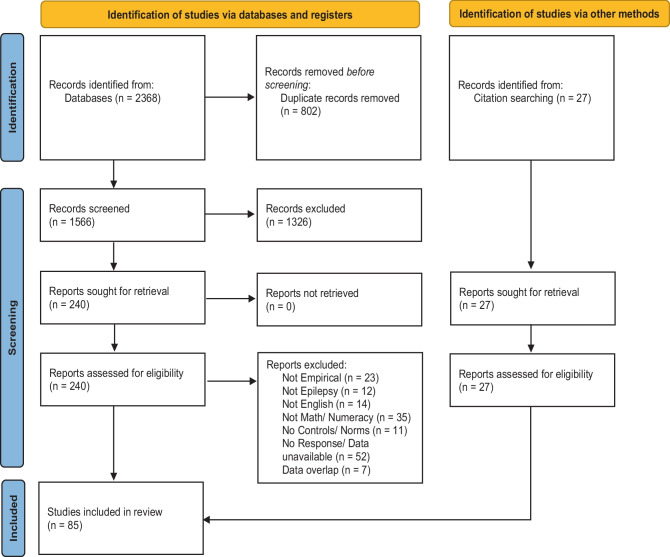
PRISMA flow diagram of study searches and selection process

A flow chart describing the process of study selection is provided in Fig. 2. The search extracted 2368 articles, with 802 duplicates. A further 27 articles were found through ancestry searches or other means (i.e. reviewing reference lists of relevant review articles).

Of the remaining 1566 articles, 1326 were excluded for not meeting inclusion criteria after reviewing titles and abstracts. A further 155 were excluded after reviewing the full-text for the following reasons: Studies (i) were not peer reviewed published empirical papers, single case studies, dissertations or conference presentations (*n* = 23); (ii) did not recruit participants with epilepsy (*n* = 12); (iii) were not published in the English language (*n* = 14); (iv) did not measure mathematics or numeracy, or included tasks not eligible for inclusion (*n* = 35); (v) did not have a control group or use normative data to calculate an effect size (*n* = 11); (vi) did not publish or provide data (via e-mail request) on the separate scores of mathematics or numeracy scores from other academic results (e.g. papers that published composite academic scores or did not provide values in order to calculate an effect size) (*n* = 52); and (vii) reported overlapping participant data with a published study that was included in the meta-analysis (*n* = 7).

### Study Characteristics

Studies were divided into adult and child groups based on the demographic sample of the epilepsy group. There were 20 adult and 67 child studies. Two studies reported on child and adult outcomes separately, thus the adult data was included in the adult meta-analysis, and the child data from the same paper was included in the child meta-analysis.

#### Adult Studies

The study characteristics of the 20 adult studies included in this review are shown in Table 1. All studies were cross-sectional. Seven studies included a control group and the results of the remaining 13 studies that did not include a control group were compared with normative data.

**Table 1:** Study characteristics – adult studies

Author (year)	Study Design	Sample	Age at Testing: mean (SD) in years	Age of onset: mean (SD) in years	Duration of epilepsy: mean (SD) in years
Abarrategui et al. (2018)	Cross-Sectional	IGE n = 61; 44.3% male Controls n = 21; 42.9% male	IGE: 32.3 (9.7) Controls: 33.2 (9)	13	18
Bornstein et al. (1988)	Cross-Sectional	Epilepsy n = 107; 50.47% male	31.2 (8.7)	-	-
Botez al. (1989)	Cross-Sectional	Epilepsy normal CT n = 31; CBS atrophy n = 33;	CT: 41.2 (2.43); CBS: 39.6 (2.16)	-	CT: 10.75 (2.26); CBS: 18.17 (2.17)
Breier et al. (2000)	Cross-Sectional	LTLE n = 27; 58% male RTLE n = 24; 30% male	LTLE: 34.7 (10); RTLE: 37 (10.2)	LTLE: 14.9 (14.9); RTLE:13.7 (13.7)	LTLE: 19.8 (12.3); RTLE: 23.3 (12.4)
Choi et al. (2011)	Cross-Sectional	Epilepsy n = 95; 40% male	39.1 (13.6)	-	-
Coimbra et al. (2006)	Cross-Sectional	Mesial TLE-HS n = 71; 47.78% male	35.2 (9.7)	-	23.9 (9.1)
Davies et al. (1995)	Cross-Sectional	LTLE n = 51; 54.72% male RTLE n = 32; 53.12% male	LTLE: 30.5; RLTE: 30.3	LTLE: 10.5; RTLE: 11.8	-
Delazer et al. (2004)	Cross-Sectional	RTLE n = 13 LTLE n = 15 Controls n = 55	RTLE: 33.5 (13.6); LTLE: 41.8 (10.6) Controls: 34.4 (8.2)	RTLE: 15.1 (14.1); LTLE: 23 (17.2)	RTLE: 18.3 (14.8); LTLE: 18.3 (14.2)
Forceville et al. (1992)	Cross-Sectional	Epilepsy n = 56; 62.5% male	31.5 (10.7)	-	-
Fowler et al. (1980)	Cross-Sectional	Epilepsy n = 118; 55.08% male	19.7 (3.71)	-	-
Levav et al. (2002)	Cross-Sectional	JME n = 11; 18.18% male TLE n = 30; 3.33% male Controls n = 55; 45% male	JME: 36.8 (6.2) TLE: 34.6 (6.9) Controls: 25.98 (16.9)	JME: 15.9 TLE: 14.5	-
Licchetta et al. (2018)	Cross-Sectional	Sleep Related Hypermotor Epilepsy n = 60; 46.67% male	38.23 (12.43)	12.63 (8.15)	
Martin et al. (2002)	Cross-Sectional	Complex Partial Seizures n = 42; 30.95% male	34.8 (11.3)	-	17 (12.1)
Pascalicchio et al. (2007)	Cross-Sectional	JME n = 50; 50% male Controls n = 50; 50% male	JME: 26.2 (7.4) Controls: 26.3 (7.45)	-	13.8 (8.51)
Seidenberg et al. (1981)	Cross-Sectional	Epilepsy Seizure Improved n = 22; 54.55% male Epilepsy Seizures Unimproved n = 25; 48% male	Improved: 22.3 (6.1); Unimproved: 21.8 (6.3)	Improved: 8.6 (6.4); Unimproved: 10 (4.6)	-
Strutt et al. (2011)	Cross-Sectional	LTLE n = 25	35 (11.4)	18.7 (8.75)	15.9 (10.8)
Tan et al. (2020)	Cross-Sectional	LTLE n = 20; 35% male RTLE n = 26; 50% male Controls n = 33; 42.4% male	LTLE: 32.8 (8.7); RTLE − 25.2 (7.4); Controls; 33.2 (11.8)	15.55	10
Thomas et al. (2014)	Cross-Sectional	JME n = 60; 25% male	31	12	21
Traianou et al. (2019)	Cross-Sectional	OLE/PLE n = 14; 57.14% male Controls n = 14 57.14% male	OLE/PLE: 32 (10.9); Controls: 32.2 (11)	12	-
Wang et al. (2018)	Cross-Section	Focal epilepsy n = 96; 53.1% male Controls n = 96; 44.8% male	Epilepsy: 34.11 (13.87) Controls: 34.45 (14.43)	-	-

*Epilepsy (Mixed Epilepsy)* epilepsy sample made up of two or more epilepsy syndromes or types of seizure, *Epilepsy with normal CT* epilepsy with normal scans on computerized tomography scans, *Epilepsy with CBS atrophy* epilepsy with cerebellar and brain stem atrophy on scans), *GGE* generalized genetic epilepsy (previously known as Idiopathic Generalized Epilepsy [IGE] which is made up of the following diagnoses: *JAE* Juvenile Absence Epilepsy, *CAE* Childhood Absence Epilepsy, *JME* Juvenile Myoclonic Epilepsy), Focal Epilepsies include: *TLE* temporal lobe epilepsy (RTLE and LTLE indicates focus to the right or left hemisphere respectively, *Mesial TLE-HS* Mesial TLE related to Hippocampal Sclerosis, *OLE* Occipital Lobe Epilepsy, *PLE* Parietal Lobe Epilepsy

There were 1215 adults with epilepsy across studies: 334 with TLE (including 138 with left TLE and 95 with right TLE); 14 with Extra-TLE/FLE; and 182 with IGE or GGE. There were no studies with an FLE group. The remaining 685 participants from 9 studies reported epilepsy samples with multiple types of seizure focus or syndromes.

The mean age of adult participants was 32.68 (*SD* = 5.71; range = 19.7–41.8 years). The mean age of epilepsy onset was 13.37 (*SD* = 3.51; range = 8.6–23.0 years) and the mean duration of epilepsy was 17.56 (*SD* = 4.21; range = 10.0–23.9 years).

#### Child Studies

The study characteristics of the child studies are shown in Table 2. Of the 67 child studies, 59 were cross-sectional, 7 were longitudinal, and 1 was a randomized control trial (RCT) design. Thirty-six studies included a control group and the results of the remaining 31 studies without a control group were compared with normative data.

**Table 2:** Study characteristics – child studies

Author (year)	Study Design	Sample	Age at Testing: mean (SD) in years	Age of onset: mean (SD) in years	Duration of epilepsy: mean (SD) in years
Adewuya et al. (2006)	Cross-Sectional	Epilepsy n = 73; 63% male Controls n = 82; 63% male	Epilepsy: 14.47 (2.1); Controls: 14.47 (2.1)	5.8 (2.36)	8.67 (3.25)
Akca Kalem et al. (2019)	Cross-Sectional	Panayiotopoulos Syndrome n = 20 Gastaut Syndrome n = 20 Controls n = 20; 40% male	PS: 10.5 (1.77) GS: 10.92 (2.76) Controls: 9.79 (1.79)	-	PS: 3.2 (2.56) GS: 2.45 (1.41)
Aldenkamp et al. (1990)	Cross-Sectional	Epilepsy n = 45; 55.56% male	9.3 (1.6)	-	6.8
Aldenkamp et al. (1999)	Cross-Sectional	Epilepsy n = 24; 33.3% male Controls n = 24; 79.2% male	Epilepsy: 8.9 (1.8) Controls: 8.8 (1.5)	2.025 (3.31)	-
Aldenkamp et al. (2005)	Cross-Sectional	Partial Onset Seizures n = 176; 52.8% male GGE n = 63 Controls n = 113; 62.8% male	Epilepsy: 9.6 (3.3) Controls: 9 (2.6)	-	-
Ayaz et al. (2013)	Cross-Sectional	Rolandic Epilepsy n = 31; 58.1% male Controls n = 31; 58.1% male	RE: 10.17 (1.61) Controls: 10.16 (1.52)	8.09 (1.97)	-
Bailet and Turk (2000)	Cross-Sectional	Idiopathic Epilepsy n = 74; 46% male Controls n = 23; 52% male	Epilepsy: 9.6 (1.7) Controls: 11.2 (1.6)	-	-
Bandeira de Lima et al. (2014)	Cross-Sectional	Epilepsy n = 31 Controls n = 31	Epilepsy: 11 (2.2) Controls: 9.8 (1)	-	4.45 (2.56)
Berg et al. (2013)	Longitudinal	Epilepsy n = 108; 59% male	11.92 (2.0)	2.9 (1.7)	-
Bigel and Smith (2001)	Cross-Sectional	TLE HS n = 15 TLE TU n = 25 TLE CD n = 7 TLE HS + TU n = 8 TLE HS + CD n = 6; 49% male	TLE HS: 13.6 (3.9) TLE TU: 12.3 (3.0) TLE CD: 13.9 (3.9) TLE HS + TU: 13.6 (2.6) TLE HS + CD: 10.8 (2.5)	TLE HS: 5.3 (4.7) TLE TU: 8.5 (3.9) TLE CD: 4.4 (3.9); TLE HS + TU: 5.5 (5.1) TLE HS + CD: 4.4 (4.5)	TLE HS: 8.5 (4.6) TLE TU: 5.4 (3.1) TLE CD: 6.0 (3.4) TLE HS + TU: 9.2 (4.3) TLE HS + CD: 8.3 (4.2)
Bohac and Wodrich (2013)	Cross-Sectional	Epilepsy n = 74; 55.4% male	-	-	-
Boll et al. (1978)	Cross-Sectional	Epilepsy n = 42; Controls n = 50	Epilepsy: 11.85 (1.53) Controls: 12.19 (1.54)	-	-
Braakman et al. (2013)	Cross-Sectional	FLE n = 32; 56.25% male Controls n = 41; 46.3% male	FLE: 11.3 (1.3) Controls: 10.5 (1.5)	4.9 (2.8)	6.1 (2.8)
Buelow et al. (2012)	Cross-Sectional	Epilepsy n = 50; 52% male	12.3 (2.2)	5.2 (3.6)	7.1 (4)
Busch et al. (2015)	Cross-Sectional	Epilepsy (Young) n = 36; 47.2% male Epilepsy (Older) n = 27; 37% male	Young: 8.36 (1.38) Older: 12.63 (1.6)	Young: 5.24 (2.24) Older: 8.26 (3.18)	Young:3.12 (2.13) Older: 4.48 (3.03)
Caplan et al. (2006)	Cross-Sectional	Complex Partial Seizures n = 93; 51% male GGE n = 56; 45% male	CPS: 10.6 (2.81) GGE: 9.6 (2.47)	CPS: 5.4 (3.62) GGE: 6.6 (2.77)	-
Chapieski et al. (2011)	Cross-Sectional	Epilepsy n = 132; 49% male	12.5	6.67 (4.24)	-
Cheng et al. (2017)	Cross-Sectional	CAE n = 35; 40% male Controls n = 33; 54.5% male	CAE: 7.3 (1.3) Controls: 6.8 (1.1)	6.7 (1.3)	0.58 (0.58)
Cheng et al. (2020)	Cross-Sectional	IED n = 97; 52.5% male No IED n = 77; 49.35% male Controls n = 71; 49.29% male	IED: 10.3 (2.1) No IEDs: 10.8 (2) Controls: 10.1 (2.5)	-	-
Conant et al. (2010)	Cross-Sectional	CAE n = 16; 31.25% male Controls n = 15; 20% male	CAE: 8 (1.3) Controls: 8.6 (1.3)	-	-
D’Alessandro et al. (1990)	Cross-Sectional	Rolandic Epilepsy n = 44; 79.5% male Controls n = 9; 100% male	RE: 10.7 Controls: 11	-	-
Danguecan and Smith (2017)	Cross-Sectional	Focal Surgical n = 61; 38% male Focal Non-Surgical n = 30; 33% male	Surgical: 12.2 (4.5) Non-Surgical: 13 (3.3)	Surgical: 5.6 (4.8) Non-Surgical: 5.1 (4.1)	Surgical: 10.9 (6.9) Non-Surgical: 13.3 (5.9)
Drewel et al. (2009)	Cross-Sectional	Epilepsy n = 173; 51% male	11.74 (1.85)	6 (3.7)	-
Dunn et al. (2010)	Longitudinal	Epilepsy n = 219; 48.9% male Controls n = 131; 48.1% male	Epilepsy: 9.9 (2.5) Controls: 10.9 (2.9)	9.7 (2.5)	-
Fastenau et al. (2008)	Cross-Sectional	Epilepsy n = 164; 50.9% male	11.8 (1.8)	6.5 (3.8)	5.2 (3.9)
Fastenau et al. (2009)	Cross-Sectional	Epilepsy n = 282; 47.9% male Controls n = 147; 46.9% male	Epilepsy: 9.7 (2.5) Controls: 10.8 (2.9)	9.5 (2.5)	-
Forceville et al. (1992)	Cross-Sectional	Epilepsy n = 33; 54.5% male	22.9 (12.4)	-	-
Gaggero et al. (1992)	Cross-Sectional	Epilepsy n = 67	*range 6–14 years	-	-
Germanò et al. (2005)	Cross-Sectional	OLE n = 22; 63.64% male Controls n = 28; 64.29% male	OLE: 10.1 (3.3) Controls: 10.9 (1.7)	4.33 (2)	-
Goldberg-Stern et al. (2010)	Cross-Sectional	BECTS n = 36 Controls n = 15	BECTS: 9.53 Controls: 11.2	-	-
Gülgönen et al. (2000)	Cross-Sectional	OLE n = 21; 61.9% male Controls n = 21; 66.67% male	OLE: 9.9 (2.96) Controls: 9.9 (2.96)	-	-
Hande Sart et al. (2006)	Cross-Sectional	Epilepsy n = 30; 70% male Controls n = 30; 70% male	Epilepsy: 10.8 (2.06) Controls: 10.8 (2.05)	7.53 (2.18)	-
Hermann et al. (2008)	Cross-Sectional	Epilepsy with comorbidities(+)* n = 28; 60.7% male No comorbidities (-) n = 24; 50% male Controls n = 48; 44.7% male	Epilepsy+: 12.3 (3.4) Epilepsy-: 12.7 (2.8) Controls: 12.7 (3)	Epilepsy+: 10.9 (3.8) Epilepsy-: 11.8 (2.9)	-
Hernandez et al. (2003)	Cross-Sectional	FLE n = 16; 75% male TLE n = 8; 50% male GGE n = 8; 50% male	FLE: 11.34 (2.77) TLE:12.44 (2.81) GGE: 11.15 (2.89)	FLE: 7.77 (3.07) TLE: 9.06 (3.5) GGE: 8.21 (3.54)	FLE: 3.82 (3.76) TLE: 3.64 (2.17) GGE: 3.84 (2.28)
Huberty et al. (1992)	Cross-Sectional	Epilepsy n = 136; 51.47% male	10.51 (1.55)	5.1 (2.92)	-
Humphries et al. (2005)	Cross-Sectional	Epilepsy n = 55; 52.7% male	9.58	4.5	-
Jackson et al. (2013)	Cross-Sectional	ILRE n = 53; 54.72% male IGE n = 41; 46.3% male Controls n = 72; 49% male	BECTS: 10.25 (1.4) Focal: 11.82 (2.94) JME: 14.62 (3.06) JAE/CAE: 12.24 (3.46) Controls: 12.86 (3.2)	BECTS: 9 (2.41) Focal: 10.51 (2.81) JME: 13.21 (4.09) JAE/CAE: 11.2 (3.52)	BECTS: 0.6 (0.34) Focal: 0.69 (0.3) JME: 0.71 (0.29) JAE/CAE: 0.775 (0.26)
Jones et al. (2010)	Longitudinal	Epilepsy Average IQ n = 41; 48.78% male Epilepsy Below Average IQ n = 23; 52.17% male Controls n = 26; 44.44% male	Epilepsy Average IQ: 9.08 (2.29) Below Average IQ: 9.95 (1.78) Controls: 10 (2.08)	Epilepsy Average IQ: 5.94 (2.77) Below Average IQ: 6.1 (2.8)	Epilepsy Average IQ: 3.14 (2.5) Below Average IQ: 3.87 (2.36)
Katzenstein et al. (2007)	Cross-Sectional	Epilepsy n = 125; 50.4% male	11.9 (1.9)	6.5 (3.8)	-
Kolfen et al. (2001)	Cross-Sectional	Epilepsy with seizures n = 37; 56.8% male Epilepsy no ASMs n = 39; 56% male Controls: 37	Seizures: 10.83 No ASMs: 9.67	-	-
Levav et al. (2002)	Cross-Sectional	CAE n = 24; 39.29% male Controls n = 55; 45% male	CAE: 14 (10.5) Controls: 25.98 (16.9)	5.6	-
Lopes et al. (2013)	Cross-Sectional	FLE n = 30; 77% male CAE n = 30; 30% male BECTS n = 30; 33% male Controls n = 30; 50% male	FLE: 10.13 (2.73) CAE: 9.93 (2.54) BECTS: 9.77 (2.43) Controls: 10.13 (2.73)	FLE: 6.4 (3.1) CAE: 6.83 (2.32) BECTS: 6.77 (2.43)	-
Lopes et al. (2014)	Cross-Sectional	Panayiotopoulos Syndrome n = 19; 76.19% male	9.11 (2.26)	5.37 (1.21)	-
Mankinen et al. (2014)	Cross-Sectional	TLE n = 21; 47.62% male Controls n = 21; 47.62% male	TLE: 11.7 Controls: 11.7	-	2.5
Martin et al. (2016)	Longitudinal	LTLE n = 16; 66% male RTLE n = 12; 42% male FLE n = 10; 50% male OLE/PLE n = 8; 63% male Non-Surgical n = 10; 60% male	LTLE: 14.6 (2.7): RTLE: 12.9 (3.2) FLE: 13.2 (3) OLE/PLE: 13.3 (2.7) Non-Surgical: 12.7 (2.4)	LTLE: 8.1 (4.1) RTLE: 8.2 (4.4) FLE: 7.4 (4.8) OLE/PLE: 5.8 (3.1) Non-Surgical: 6.3 (4.5)	-
Masur et al. (2013)	RCT	CAE n = 336	*range 6–13	-	-
Melbourne Chambers et al. (2014)	Cross-Sectional	Epilepsy n = 33; 67% male Controls n = 33; 67% male	Epilepsy: 9.6 (1.7) Controls: 9.5 (1.7)	5.5 (1.7)	-
Miranda and Smith (2001)	Cross-Sectional	RTLE n = 25; 40% male LTLE n = 25; 44% male	RTLE: 13.37 (3.32) LTLE = 13.36 (3.44)	RTLE: 6.88 (4.94) LTLE: 5.27 (4.31)	RTLE: 6.49 (4.45) LTLE: 8 (4.55)
Ng and Hodges (2020)	Cross-Sectional	Epilepsy n = 46; 43.48% male	12.34 (3.08)	6.84 (3.68)	-
Nicolai et al. (2012)	Cross-Sectional	Epilepsy n = 188; 53.7% male Controls n = 41; 61% male	Epilepsy: 10 (2.8) Controls: 10.5 (2.6)	-	-
Northcott et al. (2005)	Cross-Sectional	Rolandic Epilepsy n = 42; 61.9% male	8.5	-	-
O’Leary et al. (1981)	Cross-Sectional	Epilepsy (early onset) n = 24; 61.9% male Epilepsy (late onset) n = 24; 62.5% male	Early Onset: 12.16 (1.7) Late Onset: 12.88 (1.84)	Early Onset: 2.375 (1.625) Late Onset: 8.49 (2.6)	Early Onset: 9.57 (2.975) Late Onset: 4.475 (2.19)
O’Leary et al. (2006)	Cross-Sectional	Epilepsy n = 32 Controls n = 32	*range 6–16 years	7.3	-
Puka et al. (2015)	Longitudinal	Epilepsy with seizures n = 49; 45% male Epilepsy without seizures n = 87; 46% male	Seizures: 12.5 (3.7) No Seizures: 12.3 (3.6)	Seizures: 6.69 (4.1) No Seizures: 6.6 (4.6)	Seizures: 7.5 (4.2) No Seizures: 6.4 (4.2)
Reilly et al. (2014)	Cross-Sectional	Epilepsy n = 65; 51% male	10.8	5.65	5.1
Riva et al. (2007)	Cross-Sectional	BECTS n = 24; 66.67% male Controls n = 16; 68.75% male	BECTS: 9.42 Controls: 10	7	-
Rodin et al. (1986)	Longitudinal	Epilepsy n = 64; 51.6% male	10 (3.1)	-	3.7 (3.4)
Schoenfeld et al. (1999)	Cross-Sectional	Epilepsy n = 57; 33.3% male Controls n = 27; 48.15% male	Epilepsy: 10.78 (2.18) Controls: 11.67 (2.53)	6.39 (3.15)	-
Schwartz and Dennerll (1970)	Cross-Sectional	Epilepsy n = 82; 65.38% male Controls n = 26; 62.2% male	Epilepsy: 12.12 (2.13) Controls: 12.27 (2.25)	-	-
Sinclair et al. (2005)	Longitudinal	OLE/PLE n = 12; 46.7% male	12.14	-	-
Singhi et al. (1992)	Cross-Sectional	IGE n = 50; 64% male Controls n = 30; 63.3% male	IGE: 10 (2.4) Controls: 9.9 (2.4)	7.2 (3.02)	2.9 (1.87)
Smith et al. (2002)	Cross-Sectional	Epilepsy Surgical n = 30; 50% male Non-Surgical n = 21; 52.4% male	Surgical: 13.25 (2.99) Non-Surgical: 13.02 (3.21)	Surgical: 6.67 (3.71) Non-Surgical: 5.38 (4.7)	-
Vermeulen et al. (1994)	Cross-Sectional	Epilepsy n = 65; 61.5% male	10.3 (1.3)	5.25	5
Williams et al. (2001)	Cross-Sectional	Epilepsy n = 65; 43% male	10.42 (1.67)	-	-
Wirrell et al. (2008)	Cross-Sectional	BECTS n = 6; 83.3% male	9.1 (1.5)	8.6 (1.6)	-
Yıldız-Çoksan et al. (2019)	Cross-Sectional	Absence n = 19; 31.58% male Controls n = 19; 31.58% male	Absence: 11.25 Controls: 9.83	*range 3 months – 12 years	*range 4 months – 12 years
Zhang et al. (2020)	Cross-Sectional	BECTS n = 61; 54.1% male Controls n = 60; 50% male	BECTS: 10.81 (2.43) Controls: 10.62 (1.71)	7.36 (2.97)	-

*Epilepsy (Mixed Epilepsy)* epilepsy sample made up of two or more epilepsy syndromes or types of seizure focus (Epilepsy with comorbidities* = mixed seizure sample with (+) or without (-) comorbid psychiatric diagnoses), *GGE* generalized genetic epilepsy (previously known as Idiopathic Generalized Epilepsy [IGE] which is made up of the following diagnoses, *JAE* Juvenile Absence Epilepsy, *CAE* Childhood Absence Epilepsy, *JME* Juvenile Myoclonic Epilepsy), Idiopathic Localization-Related Epilepsy (ILRE) or Focal Epilepsies include: *TLE* temporal lobe epilepsy (RTLE and LTLE indicates focus to the right or left hemisphere respectively, *TLE HS* TLE with hippocampal sclerosis, *TLE TU* TLE with temporal lobe tumor, *TLE CD* TLE with cortical dysplasia (*note*. dual pathologies also present), *FLE* frontal lobe epilepsy, *OLE* Occipital Lobe Epilepsy, *PLE* Parietal Lobe Epilepsy, Other classification include: *IEDs/ No IEDs* Childhood (mixed) epilepsies with or without interictal epileptiform discharges, *BECTS* Benign Childhood Epilepsy with Centrotemporal Spikes, *ASM* anti-seizure medication

There were 5020 children with epilepsy across studies: 168 with TLE (including 41 with left TLE and 37 with right TLE); 88 with FLE; 122 with Extra-TLE/FLE; and 678 with IGE/GGE. The remaining 3964 participants from 51 studies reported epilepsy samples with multiple types of seizure focus or syndromes.

The mean age of child participants was 11.32 (*SD* = 2.03; range = 7.3–22.9 years). The mean age of epilepsy onset was 6.75 (*SD* = 2.07; range = 2.025–13.21 years) and the mean duration of epilepsy was 5.17 (*SD* = 3.02; range = 7 months – 13.3 years).

### Quality Ratings

#### Adult Studies

The results of the NOS quality assessment are presented in Table 3 for adult studies. Over half (55%) of studies adequately defined epilepsy cases across studies, with only 11 studies utilizing more than one method to determine epilepsy diagnosis. Methods used to determine epilepsy diagnosis ranged from using electroencephalography (EEG) or imagining results, clinical history and assessment by a neurologist against diagnostic criteria. A large number (*n* = 16; 80%) of studies recruited epilepsy participants using consecutive referrals, reducing the risk of selection bias. Of the 7 studies that used a control group for comparison, only 2 indicated that controls were recruited from the same community as cases. The remaining studies either did not provide sufficient details regarding the recruitment of controls, or retrospectively selected controls from hospital databases or subset within a normative sample. Only 4 studies reported that the control groups were neurologically healthy without a history of epilepsy or seizures. Of the 7 studies that had used a control group for comparisons, 5 matched groups on age and an additional factor (e.g. gender, FSIQ, SES). All 20 studies used at least one validated measure of mathematics or numeracy or provided a well-defined description of the experimental task. All studies that used a control group for comparisons (*n* = 7) used the same measure across groups. No studies included in this review reported the non-response rate for epilepsy or control groups.

**Table 3:** Mathematics outcomes and quality ratings (risk of bias) – adult studies

Author (year)	Epilepsy Type	Math Category and Outcomes	Main Findings	Other Factors	Quality Rating: Risk of Bias^a^		
					Selection (max 4 stars)^b^	Comparability (max 2 stars)^c^	Outcome (max 3 stars)^d^
Abarrategui et al. (2018)	IGE	Reasoning: Arithmetic (WAIS-III)	IGE vs. Controls: Significant IGE < Controls	No sig correlation with ASM dose	★★	★★	★★
Bornstein et al. (1988)	Mixed Epilepsy	Reasoning: Arithmetic (WAIS-R)	Epilepsy vs. Controls: Significant Epilepsy < Norms	-	1/2	N/A	1/1
Botez al. (1989)	Epilepsy Normal CT; ABS Atrophy	Reasoning: Arithmetic (Ottawa- Wechsler Scale)	Normal CT vs. ABS Atrophy: Significant ABS < CT	-	1/2	N/A	1/1
Breier et al. (2000)	LTLE; RTLE	Calculation: Arithmetic (WRAT-R)	LTLE vs. RTLE: Significant RTLE < LTLE; Not compared to norms	-	2/2	N/A	1/1
Choi et al. (2011)	Mixed Epilepsy	Reasoning: Numeracy Scale - word problems (Experimental)	Epilepsy vs. Norms: Significant Epilepsy < Norms	Higher FSIQ and Education correlated with better numeracy performance	0/2	N/A	1/1
Coimbra et al. (2006)	Mesial TLE- HS	Reasoning: Arithmetic (WAIS-R)	Results not compared to norms	-	2/2	N/A	1/1
Davies et al. (1995)	LTLE; RTLE	Reasoning: Arithmetic (WAIS)	LTLE vs. RTLE: Not Significant;	Sig improvement in scores 1-year post surgery for LTLE only	2/2	0	★★
Delazer et al. (2004)	RTLE; LTLE	Early Numeracy: Number Scale Calculation: Mental Calculation (Experimental)	RTLE and LTLE vs. Controls Significant RTLE and LTLE < Controls in all tasks	-	★★	★★	★★
Forceville et al. (1992)	Mixed Epilepsy	Reasoning: Arithmetic (WAIS)	Results not compared to norms	-	0/2	N/A	1/1
Fowler et al. (1980)	Mixed Epilepsy	Reasoning: Arithmetic (WAIS)	Epilepsy vs. Norms: Significant Epilepsy < Norms	-	1/2	N/A	1/1
Levav et al. (2002)	JME; TLE	Reasoning: Arithmetic (Weschler)	IGE vs. Controls: Significant IGE < Controls. No group differences within epilepsy groups	-	★★★	0	★
Licchetta et al. (2018)	Sleep-related Hypermotor Epilepsy	Reasoning: Arithmetic (WAIS-R)	Epilepsy performed the worst in arithmetic subtest	-	2/2	N/A	1/1
Martin et al. (2002)	Complex Partial Seizures	Reasoning: Arithmetic (WAIS-III)	Results not compared to norms	-	2/2	N/A	1/1
Pascalicchio et al. (2007)	JME	Reasoning: Arithmetic (WAIS-III)	JME vs. Controls: Significant JME < Controls	-	★★★★	★★	★★
Seidenberg et al. (1981)	Mixed Epilepsy (Seizures Improved vs. Unimproved)	Reasoning: Arithmetic (WAIS)	Results not compared to norms	-	1/2	N/A	1/1
Strutt et al. (2011)	LTLE	Reasoning: Arithmetic (WAIS-III)	Results not compared to norms	Arithmetic correlated with age of onset	2/2	N/A	1/1
Tan et al. (2020)	LTLE; RTLE	Reasoning: Arithmetic (WAIS-RC)	TLE vs. Controls: Significant LTLE < Controls; RTLE < Controls	-	★★	0	★★
Thomas et al. (2014)	JME	Reasoning: Arithmetic (WAIS-III)	JME vs. Norms: Significant JME < Norms	-	1/2	N/A	1/1
Traianou et al. (2019)	Extra-TLE/FLE	Mathematics: Arithmetic (Luria-Nebraska)	No significant differences Extra-TLE/FLE vs. Controls	Greater number of ASMs significantly correlated with poorer arithmetic results	★	★★	★★
Wang et al. (2018)	Focal epilepsy	Reasoning: Arithmetic (WAIS-RC)	Epilepsy vs. Controls: Significant Epilepsy < Controls	-	★★	★★	★★

*ASM* anti-seizure medication, *Norms* normative data sample, *Epilepsy (Mixed Epilepsy)* epilepsy sample made up of two or more epilepsy syndromes or types of seizure focus, *Epilepsy with normal CT* epilepsy with normal scans on computerized tomography scans, *Epilepsy with CBS atrophy *epilepsy with cerebellar and brain stem atrophy on scans), *GGE* generalized genetic epilepsy (previously known as Idiopathic Generalized Epilepsy [IGE] which is made up of the following diagnoses: *JAE* Juvenile Absence Epilepsy, *CAE* Childhood Absence Epilepsy, *JME* Juvenile Myoclonic Epilepsy), Focal Epilepsies include: *TLE* temporal lobe epilepsy (RTLE and LTLE indicates focus to the right or left hemisphere respectively, *Mesial TLE-HS* Mesial TLE related to Hippocampal Sclerosis, Extra-TLE/FLE include: *OLE* Occipital Lobe Epilepsy, *PLE* Parietal Lobe Epilepsy, *WAIS* Weschler Adult Intelligence Scale (including Revised version (R), version III and Chinese version (RC), *WRAT-R* Wide Range Achievement Test – Revised, *FSIQ* full scale intelligence quotient [IQ], *norms* normative data from assessment battery

^a^NOS quality ratings – studies with higher quality and lower risk of bias have a greater number of stars within each category. One star was allocated for each of the following criteria for *Selection*: (i) epilepsy diagnosis adequately defined, (ii) representativeness of sample, (iii) described selection of controls, (iv) definition of controls provided; a maximum of 4 stars can be awarded. One star was allocated for each of the following criteria for *Comparability*: (i) age at testing controlled in study, (ii) any for other factor controlled in study; with a maximum of two stars awarded. One star was allocated for each of the following criteria for *Outcome*: (i) ascertainment of outcome measuring one aspect of numeracy or mathematics, (ii) same method used for cases and controls, (iii) reported same non-response rate for cases and controls; with a maximum of three stars awarded

^b^In some studies, there was no control group and normative data was used instead. For studies with no control group a maximum of two (instead of 4) stars was allocated for *Selection*: (i) epilepsy diagnosis adequately defined, and (ii) representativeness of sample

^c^In some studies, there was no control group and normative data was used instead, thus no comparison was utilized. Studies with no control group received an N/A (not applicable) for this category

^d^For studies that did not have a control group a maximum of one star (instead of 3) could be awarded for this category: (i) ascertainment of outcome measuring one aspect of numeracy or mathematics

#### Child Studies

The results of the NOS quality assessment are presented in Table 4 for child studies. Approximately half (52.2%) of included studies clearly defined epilepsy cases, with 35 studies utilizing more than one method to determine epilepsy diagnosis using methods described previously. Most studies recruited epilepsy participants using consecutive referrals, reducing the risk of selection bias (*n* = 55; 82.1%). Of the 36 studies that used a control group for comparison, 27 recruited from the same community as cases. The remaining studies either did not provide sufficient details regarding the recruitment of controls, or retrospectively selected controls from hospital databases or subset within a normative sample. Less than half of studies reported control groups as neurologically healthy without a history of epilepsy or seizures (*n* = 26). Of the 36 studies that used a control group, more than half of studies matched groups on age (*n* = 21), and of those studies, 18 matched groups on an additional factor (e.g. gender, FSIQ, SES). Most studies used at least one validated measure of mathematics or numeracy or provided a well-defined description of the experimental task (*n* = 65). The other two studies used grade based or academic performance tasks. All studies with a control group used the same measure across groups (*n* = 36). Only three studies included in this review reported the non-response rate for the epilepsy or control groups.

**Table 4:** Mathematics outcomes and quality ratings (risk of bias) – child studies

Study	Epilepsy Type	Math Category and Outcomes	Findings	Other Factors	Quality Rating: Risk of Bias^a^		
					Selection (max 4 stars)^b^	Comparability (max 2 stars)^c^	Outcome (max 3 stars)^d^
Adewuya et al. (2006)	Mixed Epilepsy	Reasoning: Arithmetic (WISC-R)	Epilepsy vs. Controls: Significant Epilepsy < Controls	Sz frequency, onset, and duration associated with poor school performance (including mathematics)	★★★	★★	★★
Akca Kalem et al. (2019)	Panayiotopoulos Syndrome & Gastaut Syndrome	Reasoning: Arithmetic (WISC-R)	PS and GS vs. Controls: Significant PS and GS < Controls	-	★★★	★★	★★
Aldenkamp et al. (1990)	Mixed Epilepsy	Reasoning: Arithmetic (WISC-R)	Not significance tested with norms	-	1/2	N/A	1/1
Aldenkamp et al. (1999)	Mixed Epilepsy	Calculation: Arithmetic (Groninger School Onderzoek)	No significant differences	-	★★★	★★	★★
Aldenkamp et al. (2005)	Partial Onset Seizures & GGE	Calculation: Arithmetic (Dutch Short Screening Test)	Partial sz and GGE vs. Controls: Significant Partial sz and GGE < Controls	-	★★	0	★★
Ayaz et al. (2013)	Rolandic Epilepsy	Reasoning: Arithmetic (WISC-R)	No significant differences	-	★★★	★★	★★
Bailet and Turk (2000)	Idiopathic epilepsy	Calculation: Arithmetic (WRAT-R)	Epilepsy vs. Controls: Significant Epilepsy < Controls	No correlation with sz onset or frequency; Sig differences in arithmetic outcomes with ASM type	★★	0	★★
Bandeira de Lima et al. (2014)	Mixed Epilepsy	Mathematics: Mathematics (Academic Performance Test)	No significant differences	-	★★	0	★★
Berg et al. (2013)	Mixed Epilepsy	Calculation: Arithmetic (WRAT)	No significant differences	FSIQ correlated with arithmetic scores; ASM medication correlated with lower arithmetic scores	1/2	N/A	1/1
Bigel and Smith (2001)	TLE	Calculation: Arithmetic (WRAT)	Not significance tested with norms	-	0/2	N/A	1/1
Bohac and Wodrich (2013)	Mixed Epilepsy	Mathematics: Mathematics (TerraNova Comprehensive Test of Basic Skills)	Not significance tested	Higher sz frequency related to poorer math achievement	1/2	N/A	1/1
Boll et al. (1978)	Mixed Epilepsy	Mathematics: Mathematics (Peabody Individual Achievement Test)	Epilepsy vs. Controls: Significant Epilepsy < Controls	-	★	0	★★
Braakman et al. (2013)	FLE	Mathematics: Arithmetic (Tempotest)	FLE vs. Controls: Significant FLE < Controls	-	★★★	★	★
Buelow et al. (2012)	Mixed Epilepsy	Calculation: Calculation (WCJ-R)	Epilepsy vs. Norms: Significant Epilepsy < Norms	-	1/2	N/A	1/1
Busch et al. (2015)	Mixed Epilepsy	Calculation: Calculation and Math Fluency (WCJ-III) Reasoning: Applied Problems (WCJ-III)	Epilepsy < Norms (not impaired; not significance tested)	-	2/2	N/A	1/1
Caplan et al. (2006)	Complex Partial Seizures & GGE	Reasoning: Mathematics Reasoning (WIAT)	Not significance tested; no impairment both groups	-	1/2	N/A	1/1
Chapieski et al. (2011)	Mixed Epilepsy	Mathematics: Math Composite (K-TEA-II)	Not significance tested with norms; not impaired	FSIQ significantly correlated with math scores	0/2	N/A	1/1
Cheng et al. (2017)	CAE	Early Numeracy: Number comparison task and Simple subtraction (OPES)	CAE vs. Controls: Significant CAE < Norms both math outcomes	Number comparison task correlated with intelligence and WM; not correlated with attention or executive function	★★★	★★	★★
Cheng et al. (2020)	Mixed Epilepsy	Early Numeracy: Simple subtraction (OPES)	Epilepsy vs. Controls: Significant Epilepsy with interictal epileptiform discharges < Controls	-	★★★	★★	★★
Conant et al. (2010)	CAE	Mathematics: Mathematics (WRAT-III)	CAE vs. Controls: Not Significant	-	★★★	★	★★★
D’Alessandro et al. (1990)	Rolandic Paroxysmal Epilepsy	Reasoning: Arithmetic (WISC)	Epilepsy vs. Controls: Not Significant	-	★★★	0	★★
Danguecan and Smith (2017)	Focal epilepsy	Calculation: Arithmetic (Various tests)	Not significance tested with norms	Presence of sz associated with poor WM, which led to poor arithmetic; number of ASMs did not predict additional variance beyond WM	1/2	N/A	1/1
Drewel et al. (2009)	Mixed Epilepsy	Mathematics: Mathematics (WCJ – R)	Not significance tested with norms; not impaired	-	1/2	N/A	1/1
Dunn et al. (2010)	Mixed Epilepsy	Mathematics: Mathematics (WCJ – R)	Epilepsy vs. Sibling Controls: Not significant	-	★★	★	★★★
Fastenau et al. (2008)	Mixed Epilepsy	Calculation: Calculation (WCJ – R) Reasoning: Applied Problems (WCJ – R)	Not significance tested with norms	Earlier age of onset greater risk for poor math calculation outcomes	2/2	N/A	1/1
Fastenau et al. (2009)	Mixed Epilepsy	Calculation: Calculation (WCJ – R)	Epilepsy vs. Sibling controls: Not significant	-	★★	0	★★
Forceville et al. (1992)	Mixed Epilepsy	Reasoning: Arithmetic (WISC-R)	Results not compared to norms	-	0/2	N/A	1/1
Gaggero et al. (1992)	Mixed Epilepsy	Reasoning: Arithmetic (WISC)	Epilepsy vs. Norms controls: Not significant	-	1/2	NA	1/1
Germanò et al. (2005)	Childhood Epilepsy with Occipital Paroxysms	Calculation: Calculation (ABCA) Early Numeracy: Counting, Number Dictation, and Number fact retrieval (ABCA)	Epilepsy impaired in all outcomes except counting	-	★★★★	0	★★
Goldberg-Stern et al. (2010)	BECTS	Mathematics: Mathematics (K-ABC)	No significant differences	-	★★★	0	★★
Gülgönen et al. (2000)	OLE	Reasoning: Arithmetic (WISC-R) Calculation: Written arithmetic (WRAT-III)	OLE vs. Controls: Significant OLE < Controls in mental arithmetic only	-	★★★★	★★	★★
Hande Sart et al. (2006)	Mixed Epilepsy	Calculation: Arithmetic (WRAT-III)	Epilepsy vs. Controls: Significant Epilepsy < Controls	-	★★★★	★★	★★
Hermann et al. (2008)	Mixed Epilepsy with/ without psychiatric comorbidities	Calculation: Arithmetic (WRAT-III)	Epilepsy with comorbidities vs. Controls: Significant Epilepsy < Controls	-	★★★★	★★	★★
Hernandez et al. (2003)	FLE; TLE; GGE	Reasoning: Arithmetic (WISC-III)	Not significance tested with norms	-	2/2	N/A	1/1
Huberty et al. (1992)	Mixed Epilepsy	Mathematics: Mathematics (CAT/ ITBS)	Not significance tested with norms	Math outcomes not correlated with age of onset or sz frequency	2/2	N/A	1/1
Humphries et al. (2005)	Mixed Epilepsy	Calculation: Calculation (WCJ-R) Reasoning: Applied Problems (WCJ-R)	Not significance tested with norms	-	0/2	N/A	1/1
Jackson et al. (2013)	ILRE; IGE	Calculation: Arithmetic (WRAT-III)	ILRE; GGE vs. Controls: Significant ILRE and GGE < Controls	-	★★★	★★	★★
Jones et al. (2010)	Mixed Epilepsy	Reasoning: Mathematics Reasoning (WIAT Screener)	Epilepsy vs. Controls: Significant Epilepsy with below average IQ < Controls	No difference in math outcome according to IQ; Math performance declined at follow up despite improved sz frequency	★★★★	0	★★
Katzenstein et al. (2007)	Mixed Epilepsy	Mathematics: Broad Math Composite (WCJ-R)	Not significance tested with norms	-	1/2	N/A	1/1
Kolfen et al. (2001)	Mixed Epilepsy	Reasoning: Arithmetic (Adaptives Intelligenzdiagnostikum)	No significant differences	Seizures before 3 years of age had poorer arithmetic scores	★★★	★★	★★
Levav et al. (2002)	CAE	Reasoning: Arithmetic (Weschler)	IGE vs. Controls: Significant IGE < Controls	-	★★★	0	★
Lopes et al. (2013)	FLE; CAE; BECTS	Reasoning: Arithmetic (WISC-III)	FLE; CAE; BECTS vs. Controls: Significant: FLE < Controls only	Arithmetic performance not correlated with seizure onset, duration, sz frequency or ASMs	★★	★★	★★
Lopes et al. (2014)	Panayiotopoulos Syndrome	Reasoning: Arithmetic (WISC-III)	Not significance tested with norms	-	2/2	N/A	1/1
Mankinen et al. (2014)	TLE	Reasoning: Arithmetic (WISC-III)	No significant differences	-	★★★★	★★	★★
Martin et al. (2016)	LTLE; RTLE; FLE; OLE/PLE; Non-Surgical	Mathematics: Mathematics Composite (KTEA-II)	Not significance tested with norms	Surgery did not impact academic outcomes; non-surgical group declined at follow up	2/2	N/A	1/1
Masur et al. (2013)	CAE	Calculation: Arithmetic (WRAT-III)	Not significance tested with norms	-	1/2	N/A	1/1
Melbourne Chambers et al. (2014)	Mixed Epilepsy	Mathematics: Math Expanded (WRAT-III)	Epilepsy vs. Controls: Significant Epilepsy < Controls	-	★★★★	★★	★★★
Miranda and Smith (2001)	RTLE; LTLE	Reasoning: Arithmetic (Various tests)	Not significance tested with norms	No change in scores post-surgery	1/2	N/A	1/1
Ng and Hodges (2020)	Mixed Epilepsy	Calculation: Calculation (WCJ-IV/ WAIT-III)	Not significance tested with norms	Global ability, processing speed indexes and attentional problems correlated with math calculation; ASM not correlated with math	1/2	N/A	1/1
Nicolai et al. (2012)	Mixed Epilepsy (IED group and Seizure group)	Calculation: Arithmetic (Dutch short screening test)	IED/ Sz vs. Controls: Significant Both groups < controls	-	★★★	0	★★
Northcott et al. (2005)	Benign Rolandic Epilepsy	Reasoning: Arithmetic (WISC-III) Mathematics: Mathematics (WIAT)	Epilepsy vs. Norms: Significant Epilepsy > Norms Arithmetic subtest only	-	2/2	N/A	1/1
O’Leary et al. (1981)	Mixed Epilepsy	Reasoning: Arithmetic (WISC-R)	Not significance tested with norms	No differences in math reasoning for early vs. late onset seizures	1/2	N/A	1/1
O’Leary et al. (2006)	Mixed Epilepsy	Reasoning: Arithmetic (WISC-III)	Epilepsy vs. Controls: Significant Epilepsy < Controls	-	★	★★	★★
Puka et al. (2015)	Mixed Epilepsy	Calculation: Arithmetic (WIAT/ WRAT/ WCJ)	Not significance tested with norms	Decline in math from baseline to follow up irrespective of seizure status; temporal lobe resection improved math calculation	1/2	N/A	1/1
Reilly et al. (2014)	Mixed Epilepsy	Calculation: Math Computation (WRAT-IV)	Not significance tested with norms	Math calculation related to processing speed after controlling for IQ; Sz onset and WM not correlated with math after controlling for IQ	2/2	N/A	1/1
Riva et al. (2007)	BECTS	Reasoning: Arithmetic (WISC-R)	BECTS with Right or Left EEG focus vs. Controls: Not significant	-	★★★★	0	★★
Rodin et al. (1986)	Mixed Epilepsy	Reasoning: Arithmetic (WISC)	Not significance tested with norms	-	2/2	N/A	1/1
Schoenfeld et al. (1999)	Complex Partial Seizures	Calculation: Arithmetic (WRAT-III)	Epilepsy vs. Controls: Significant Epilepsy < Controls	Age of onset strongest predictor of academic achievement	★★★★	0	★★
Schwartz and Dennerll (1970)	Mixed Epilepsy	Reasoning: Arithmetic (WISC)	Epilepsy vs. Controls: Not Significant	-	★★	0	★★
Sinclair et al. (2005)	OLE/PLE	Calculation: Arithmetic (WRAT)	Not significance tested with norms	-	1/2	N/A	1/1
Singhi et al. (1992)	IGE	Reasoning: Arithmetic (WISC)	Epilepsy vs. Controls: Significant Epilepsy < Controls	-	★★	★★	★★
Smith et al. (2002)	Mixed Epilepsy	Calculation: Arithmetic (WIAT)	Not significance tested with norms	Seizure frequency had negative impact on arithmetic scores; no correlation with AEDs or age of onset or duration of epilepsy	2/2	N/A	1/1
Vermeulen et al. (1994)	Mixed Epilepsy	Reasoning: Arithmetic (WISC-R)	Not significance tested with norms	-	★★★	★★	★
Williams et al. (2001)	Mixed Epilepsy	Reasoning: Applied Problems (WCJ – R)	Epilepsy vs. Controls: Not Significant	-	1/2	N/A	1/1
Wirrell et al. (2008)	BECTS	Mathematics: Broad Mathematics Composite (WIAT-II)	Not significance tested with norms	Decline in math achievement scores after 6 months ASM treatment	2/2	N/A	1/1
Yıldız-Çoksan et al. (2019)	Absence Epilepsy	Mathematics: Mathematics Grades	Epilepsy vs. Controls: Not Significant	-	★★★	★★	★★
Zhang et al. (2020)	BECTS	Early Numeracy: Simple Subtraction and Number comparison (OPES)	BECTS vs. Controls: Not Significant both outcomes	Age onset < 8 years and ASM related to poor performance on all math outcomes	★★	0	★

*Epilepsy (Mixed Epilepsy)* epilepsy sample made up of two or more epilepsy syndromes or types of seizure focus (Epilepsy with comorbidities* = mixed seizure sample with (+) or without (-) comorbid psychiatric diagnoses), *GGE* generalized genetic epilepsy (previously known as Idiopathic Generalized Epilepsy [IGE] which is made up of the following diagnoses: *JAE* Juvenile Absence Epilepsy, *CAE* Childhood Absence Epilepsy, *JME* Juvenile Myoclonic Epilepsy), Idiopathic Localization-Related Epilepsy (ILRE) or Focal Epilepsies include: *TLE* temporal lobe epilepsy (RTLE and LTLE indicates focus to the right or left hemisphere respectively, *TLE HS* TLE with hippocampal sclerosis, *TLE TU* TLE with temporal lobe tumor, *TLE CD* TLE with cortical dysplasia (*note*. dual pathologies also present), *FLE* frontal lobe epilepsy, *OLE* Occipital Lobe Epilepsy, *PLE* Parietal Lobe Epilepsy, *BECTS* Benign Childhood Epilepsy with Centrotemporal Spikes, Other classification include: *IEDs/ No IEDs* Childhood (mixed) epilepsies with or without interictal epileptiform discharges, Other terms: *Sz* Seizure, *Norms* normative data from assessment battery, *FSIQ* full scale intelligence quotient [IQ], *WM* working memory, *ADHD* attention deficit hyperactivity disorder, *EEG* electroencephalogram, *ASM* anti-seizure medication, Assessment batteries include: *WISC* Weschler Intelligence Scale for Children (including revised version (R), or 3rd edition (III), *WRAT* Wide Range Achievement Test (including revised version (R), 3rd edition (III), or 4th edition (IV), *WCJ* Woodcock-Johnson (including revised version (R), 3rd edition (III), or 4th edition (IV), *WIAT* Weschler Individual Achievement Test (including 2nd edition (II), 3rd edition (III), or fourth edition (IV), *K-TEA* Kaufman Test of Educational Achievement (II – second edition), *OPES* Online Experimental Psychological System, *ABCA* ABCA test of arithmetic skills, *K-ABC* Kaufman Assessment Battery for Children, *CAT* California Achievement Test, *ITBS* Iowa Tests of Basic Skills

^a^NOS quality ratings – studies with higher quality and lower risk of bias have a greater number of stars within each category. One star was allocated for each of the following criteria for *Selection*: (i) epilepsy diagnosis adequately defined, (ii) representativeness of sample, (iii) described selection of controls, (iv) definition of controls provided; a maximum of 4 stars can be awarded. One star was allocated for each of the following criteria for *Comparability*: (i) age at testing controlled in study, (ii) any for other factor controlled in study; with a maximum of two stars awarded. One star was allocated for each of the following criteria for *Outcome*: (i) ascertainment of outcome measuring one aspect of numeracy or mathematics, (ii) same method used for cases and controls, (iii) reported same non-response rate for cases and controls; with a maximum of three stars awarded

^b^In some studies, there was no control group and normative data was used instead. For studies with no control group a maximum of two (instead of 4) stars was allocated for *Selection*: (i) epilepsy diagnosis adequately defined, and (ii) representativeness of sample

^c^In some studies, there was no control group and normative data was used instead, thus no comparison was utilized. Studies with no control group received an N/A (not applicable) for this category

^d^For studies that did not have a control group a maximum of one star (instead of 3) could be awarded for this category: (i) ascertainment of outcome measuring one aspect of numeracy or mathematics

### Study Characteristics – Mathematics Measures

The description of each mathematics measure and related findings for each study are provided in Table 3 for adult studies and Table 4 for child studies.

#### Adult Studies

##### Early Numeracy

Only one study measured early numeracy skills in adults, which was evaluated using an experimental analogue number scale task, in which participants needed to choose the position of an Arabic numeral or number-word on a scale from zero to 100 (Delazer et al., 2004).

##### Calculation

There were two studies that utilized measures that evaluate mathematics calculation abilities. One study used a subtest from a standardized assessment battery that evaluated arithmetic calculations, and one study used two experimental tasks that involved mental arithmetic calculation, where participants were required to mentally compute an answer to visually presented addition, subtraction, and multiplication questions (Delazer et al., 2004).

##### Reasoning

Mathematics reasoning was the most used measure across adult studies. Of the 17 studies that evaluated mathematics reasoning, most studies used the Arithmetic subtest from various editions of the Weschler Adult Intelligence Scale (WAIS; *n* = 16). One study used a validated 11-question scale that involved word problems (Choi et al., 2011).

##### Combined Mathematics

Combined mathematics included all numeracy, calculation, and reasoning measures from 20 studies. This also includes one study not included above, which reported a mathematics composite score of both early numeracy and calculation (Traianou et al., 2019).

#### Child Studies

##### Early Numeracy

Three studies measured early numeracy skills, two studies used two tasks involving number comparison task and simple subtraction, and one study used simple subtraction only. One study evaluated counting, number dictation and number fact retrieval.

##### Calculation

There were 24 studies that utilized measures that evaluate mathematics calculation abilities. All measures were from standardized assessment batteries. Most studies used a test of arithmetic calculation in either verbal or written forms task (*n* = 14), other studies used the test of mathematics calculation (*n* = 9), and one study used a mathematics fluency task (*n* = 1).

##### Reasoning

Mathematics reasoning was the most used measure across child studies. Of the 29 studies that evaluated mathematics reasoning, most studies used the Arithmetic subtest from various editions of the Wechsler Intelligence Scale for Children (WISC; *n* = 24), which requires speeded responses to mathematics word problems. Five studies used measures of applied problem solving or mathematics reasoning tasks (*n* = 5).

##### Combined Mathematics

Mathematics combines all numeracy, calculation, and reasoning measures from all 67 studies. This also included 16 studies not included above. Of those studies, 11 reported a mathematics composite score (e.g., combination of calculation and reasoning subtests). Five studies reported a standardized classroom-based assessment of mathematics scores (i.e., California Achievement Test, Iowa Tests of Basic Skills, Brazilian Academic Performance Test and a Dutch Educational Achievement Test) and one study reported classroom grades in mathematics.

### Meta-Analyses

Two separate meta-analyses were conducted to evaluate the magnitude and significance of the differences in mathematics outcomes in adults and children with epilepsy.

#### Adult Studies

##### Early Numeracy

Only one study measured early numeracy skills in adults and found moderate impairments in early numeracy for adults with left and right TLE (Delazer et al., 2004).

##### Calculation

Adults with epilepsy, which only included adults with TLE, performed worse than healthy adults on measures of mathematics calculation, but this was not significant (*k* = 2, *g*= -0.57, 95% CI -1.179, 0.096, *p =* .066). There was significant heterogeneity between studies (Q = 32.891, *df* = 4, *p* < .001, T = 0.631, T^2^ = 0.399, I^2^ = 87.839, 95% prediction interval cannot be computed [*k* = 2]). In relation to other sites of seizure focus, no included studies reported mathematics calculation for adults with FLE, extra-TLE/FLE or GGE.

##### Reasoning

Adults with epilepsy, pooled across all subtypes, performed significantly worse than healthy adults on measures of mathematics reasoning (*k* = 17, *g*= -0.74, 95% CI -1.016, -0.456, *p <* .001). There was significant heterogeneity between studies (Q = 325.662, *df* = 19, *p* < .001, T = 0.607, T^2^ = 0.368, I^2^ = 94.166, 95% prediction interval − 2.0644, 0.5924).

**Fig. 3:**
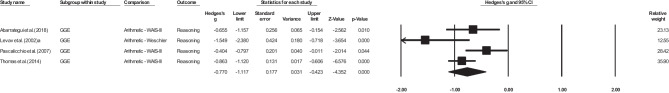
Forest plot of individual and pooled adult Genetic Generalized Epilepsy (GGE) effect sizes (Hedges’s g) with 95% confidence interval for mathematics reasoning outcomes

Further analyses according to the site of seizure focus found that, relative to healthy controls, adults with TLE (*k =* 5, *g=* -0.5, 95% -0.953, -0.052, *p =* .029), and GGE (*k* = 4, *g*= -0.77, 95% CI -1.177, -0.423, *p = <* 0.001, see Fig. 3) demonstrated significant impairments in mathematics reasoning. No studies examined mathematics reasoning in adults with FLE or extra-TLE/FLE. For TLE, there was significant heterogeneity between studies (Q = 54.319, *df* = 6, *p* < .001, T = 0.558, T^2^ = 0.312, I^2^ = 88.954, 95% prediction interval − 2.4255, 1.4195). Tests of heterogeneity were not significant for GGE (*p* > .05).

##### Combined Mathematics

**Fig. 4:**
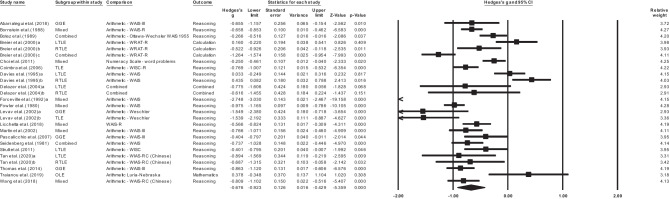
Forest plot of individual and pooled adult epilepsy effect sizes (Hedges’s g) with 95% confidence interval across all combined mathematics outcomes

Adults with epilepsy, pooled across all subtypes, performed significantly worse than healthy adults across combined mathematics tasks (*k* = 20, *g*= -0.68, 95% CI -0.923, -0.429, *p <* .001, see Fig. 4); However, there was significant heterogeneity between studies (Q = 367.214, *df* = 25, *p* < .001, T = 0.602, T^2^ = 0.362, I^2^ = 93.192, 95% prediction interval − 1.9675, 0.6155).

**Fig. 5:**
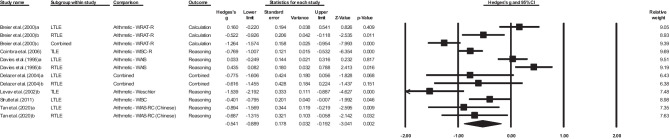
Forest plot of individual and pooled adult Temporal Lobe Epilepsy (TLE; including left, right or combined unilateral TLE) effect sizes (Hedges’s *g*) with 95% confidence interval across all combined mathematics outcomes

Further analyses according to the site of seizure focus found that, relative to healthy adults, significant impairments in mathematics were found in TLE (*k =* 7, *g=* -0.54, 95% -0.889, -0.192, *p =* .002, see Fig. 5). No studies examined additional mathematics outcomes in adults with FLE, extra-TLE/FLE or GGE. There was significant heterogeneity between studies (Q = 92.405, *df* = 11, *p* < .001, T = 0.558, T^2^ = 0.312, I^2^ = 88.096, 95% prediction interval − 2.048, 0.966).

#### Child Studies

##### Early Numeracy

Children with epilepsy, pooled across all subtypes, performed significantly worse than typically developing children on measures of early numeracy (*k* = 4, *g*= -0.38, 95% CI -0.596, -0.17, *p <* .001). Tests of heterogeneity were not significant (*p* > .05).

In relation to site of seizure focus, no studies measured early numeracy skills for children with TLE, FLE, extra-TLE/FLE. Only one study evaluated early numeracy in GGE, which found small to moderate impairments in early numeracy skills (Cheng et al., 2017).

##### Calculation

Children with epilepsy, pooled across all subtypes, performed significantly worse than typically developing children on measures of mathematics calculation, (*k* = 23, *g*= -0.76, 95% CI -0.971, -0.553, *p <* .001). There was significant heterogeneity between studies (Q = 347.452, *df* = 24, *p* < .001, T = 0.494, T^2^ = 0.244, I^2^ = 93.093, 95% prediction interval − 1.8129, 0.2889).

Further analyses according to site of seizure focus found that, relative to typically developing children, children with GGE were significantly impaired in mathematics calculation (*k* = 3, *g*= -0.7, 95% CI -1.312, -0.085, *p =* .026). No significant impairments were found in children with extra-TLE/FLE (*k* = 3, *g*= -1, 95% CI -2.086, 0.078, *p =* .069). Only one study examined mathematics calculation in TLE, and found large impairments in calculation (Bigel & Smith, 2001). No studies evaluated mathematics calculation in children with FLE. There was significant heterogeneity between studies (Q = 23.082, *df* = 2, *p* < .001, T = 0.513, T^2^ = 0.263, I^2^ = 91.335, 95% prediction interval − 8.3347, 6.9367) for GGE, and for extra-TLE/FLE (Q = 12.22, *df* = 2, *p* < .001, T = 0.868, T^2^ = 0.754, I^2^ = 83.633, 95% prediction interval − 14.0781, 12.0701).

##### Reasoning

Children with epilepsy, pooled across all subtypes, performed significantly worse than typically developing on measures of mathematics reasoning (*k* = 29, *g*= -0.57, 95% CI -0.778, -0.366, *p <* .001). There was significant heterogeneity between studies (Q = 400.791, *df* = 35, *p* < .001, T = 0.581, T^2^ = 0.338, I^2^ = 91.267, 95% prediction interval − 1.7842, 0.6402).

Further analyses according to the site of seizure focus found that, relative to typically developing children, children with TLE (*k =* 3, *g=* -0.41, 95% -0.652, -0.161, *p =* .001), FLE (*k =* 2, *g=* -0.89, 95% -1.302, -0.479, *p <* .001), extra-TLE/FLE (*k =* 3, *g=* -0.5, 95% -0.875, -0.119, *p =* .01), and GGE (*k* = 5, *g*= -0.73, 95% CI -1.137, -0.330, *p = <* 0.001) demonstrated significant impairments in mathematics reasoning. For GGE, there was significant heterogeneity between studies (Q = 11.889, *df* = 4, *p* = .018, T = 0.363, T^2^ = 0.132, I^2^ = 66.356, 95% prediction interval − 2.0624, 0.5964). Tests of heterogeneity were not significant for TLE, FLE, or extra-TLE/FLE (*p* > .05).

##### Combined Mathematics

**Fig. 6:**
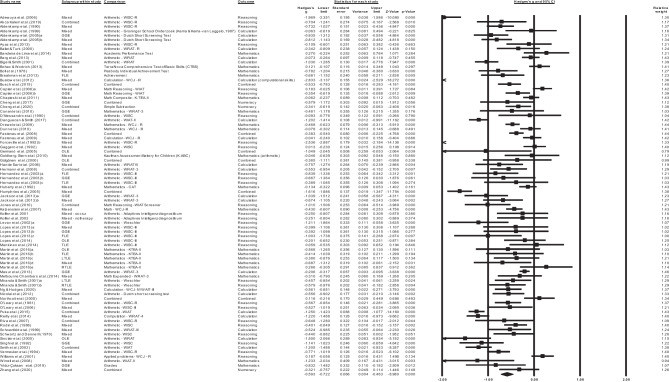
Forest plot of individual and pooled child epilepsy effect sizes (Hedges’s g) with 95% confidence interval across all combined mathematics outcomes

Children with epilepsy, pooled across all subtypes, performed significantly worse than typically developing children across mathematics tasks combined (*k* = 67, *g*= -0.59, 95% CI -0.722, -0.463, *p <* .001, see Fig. 6). However, there was significant heterogeneity between studies (Q = 1017.837, *df* = 79, *p* < .001, T = 0.544, T^2^ = 0.296, I^2^ = 92.238, 95% prediction interval − 1.6876, 0.5016).

**Fig. 7:**
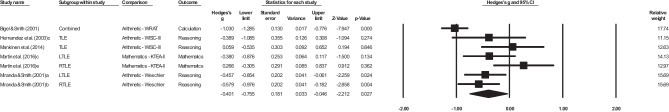
Forest plot of individual and pooled child Temporal Lobe Epilepsy (TLE) effect sizes (Hedges’s g) with 95% confidence interval across all combined mathematics outcomes

**Fig. 8:**
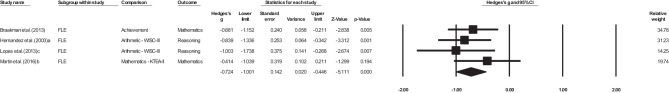
Forest plot of individual and pooled child Frontal Lobe Epilepsy (FLE) effect sizes (Hedges’s g) with 95% confidence interval across all combined mathematics outcomes

**Fig. 9:**
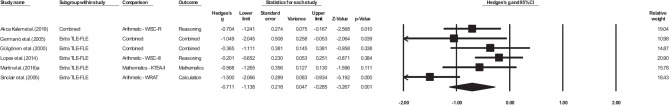
Forest plot of individual and pooled child Extra-Temporal Lobe/ Frontal Lobe Epilepsy (TLE/FLE) effect sizes (Hedges’s g) with 95% confidence interval across all combined mathematical outcomes

**Fig. 10:**
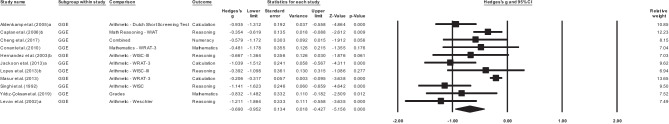
Forest plot of individual and pooled child Generalized Genetic Epilepsy (GGE) epilepsy effect sizes (Hedges’s g) with 95% confidence interval across all combined mathematics outcomes

Further analyses according to the site of seizure focus found that, relative to typically developing children, children with TLE (*k =* 5, *g=* -0.4, 95% -0.755, -0.046, *p =* .027, see Fig. 7), FLE (*k =* 4, *g=* -0.72, 95% -1.001, -0.446, *p <* .001, see Fig. 8), extra-TLE/FLE (*k =* 7, *g=* -0.71, 95% -1.138, -0.047, *p =* .001, see Fig. 9), and GGE (*k =* 11, *g=* -0.69, 95% -0.952, -0.427, *p <* .001, see Fig. 10) demonstrated significant impairments in mathematics. For TLE, there was significant heterogeneity between studies (Q = 26.197, *df* = 6, *p* < .001, T = 0.41, T^2^ = 0.168, I^2^ = 77.096, 95% prediction interval − 1.8271, 1.0251). There was also significant heterogeneity between studies for extra-TLE/FLE (Q = 13.701, *df* = 5, *p* = .018, T = 0.417, T^2^ = 0.174, I^2^ = 63.506, 95% prediction interval − 2.0924, 0.6704) and in children with GGE (Q = 44.146, *df* = 10, *p* < .001, T = 0.358, T^2^ = 0.128, I^2^ = 67.348, 95% prediction interval − 1.5544, 0.1744). Tests of heterogeneity were not significant for children with FLE (*p* > .05).

### Meta-regression of Moderator Variables

Meta-regressions were conducted with the pooled adult and child epilepsy groups, separately. For adult studies, 19 papers provided age at testing, 9 studies reported age of onset, and 8 studies provided duration of epilepsy. For child studies, 72 papers provided age at testing, 50 provided age of onset, and 21 studies provided duration of epilepsy. No significant associations were found between age at testing, age of onset, or duration of epilepsy and with calculation, reasoning, and combined mathematics for both adult and child studies (all *ps* > 0.05). An insufficient number of studies evaluated early numeracy for meta-regression.

### Systematic Review of Other Moderator Variables

#### Seizure Frequency

##### Adult Studies

No studies examined mathematics outcomes with seizure frequency in adults with epilepsy.

##### Child Studies

Nine studies examined seizure frequency in children. Of those, three studies found that higher frequency of seizures were related to poorer mathematics outcomes in children with epilepsy with mixed subtypes (Bohac & Wodrich, 2013; Reilly et al., 2014; Smith et al., 2002), and another study that found seizure frequency was related to poorer school performance, which included mathematics (Adewuya et al., 2006). One study reported a decline in mathematics reasoning outcomes in children with epilepsy and average IQ with improved seizure frequency at follow up when compared to children with no changes to seizure frequency (Jones et al., 2010). The remaining four studies found no correlation between seizure frequency with mathematics outcomes (Bailet & Turk, 2000; Huberty et al., 1992; Lopes et al., 2013; Ng & Hodges, 2020).

#### Anti-Seizure Medications (ASM)

##### Adult Studies

Two studies examined ASMs in adults. One study found a significant correlation between higher polypharmacy and poorer arithmetic results in adults with extra-TLE/FLE (Traianou et al., 2019). The second study found no correlation with mathematics reasoning and daily ASM (valproate) dose in adults with GGE (Abarrategui et al., 2018).

##### Child Studies

Nine studies examined ASMs in children, with five studies reporting a significant relationship. One study found a decline in mathematics achievement outcomes six months after commencing Sulthiame ASM treatment (Wirrell et al., 2008), and another study reported children who took valproate, compared to a mix of other medications, had poorer numeracy skills in children with benign epilepsy of childhood with central temporal spikes (BECTS) (Zhang et al., 2020). The third study reported poorer arithmetic outcomes in children who were treated with valproic acid compared to carbamazepine at baseline, however, no differences emerged between groups at second and third follow ups over five years (Bailet & Turk, 2000). The fourth study revealed children who were treated with ASM performed significantly lower than children with epilepsy who were not taking any ASM for epilepsy (Berg et al., 2013). And the final study reported poly therapy or a past history of being treated with three or more ASMs in the past, had a detrimental impact on mathematics calculation outcomes (Reilly et al., 2014).

Of the four studies that found no relationship, one study reported no correlation between ASMs and arithmetic performance in children with FLE, GGE or focal epilepsies (Lopes et al., 2013). Two studies found no relationship with ASMs and mathematics in a mixed sample of children with epilepsy (Ng & Hodges, 2020; Smith et al., 2002). The final study reported that number of ASMs did not predict additional variance in mathematics calculation after controlling for working memory (Danguecan & Smith, 2017).

#### Surgical Outcomes

##### Adult Studies

One study examined surgical outcomes in adults. This study reported a significant improvement in mathematics reasoning scores one-year post surgery for adults with left TLE, with no change in right TLE (Davies et al., 1995).

##### Child Studies

Three studies examined surgical outcomes in children, with one study reporting detrimental outcomes in mathematics calculation for children who underwent surgery for epilepsy. That study found mathematics calculation scores declined post-surgery irrespective in both seizure free and ongoing seizure groups (Puka et al., 2015). However, this study also found that temporal lobe resections were associated with higher scores in arithmetic. In contrast, no change in mathematics scores post-surgery were reported in children with right and left TLE (Miranda & Smith, 2001), and no differences emerged in mathematics calculation performance in surgical and non-surgical groups. And the final study found epilepsy surgery did not impact academic outcomes in focal epilepsies, with a decline in outcomes observed for the non-surgical group at follow up (Martin et al., 2016).

#### Cognitive Skills

##### Adult Studies

One study examined cognitive skills in adults. This study found that mathematics reasoning performance was correlated with full scale intelligence quotient (FSIQ) in a mixed epilepsy sample (Choi et al., 2011). No studies examined the relationship between working memory and mathematics outcomes in adults.

##### Child Studies

Four studies examined relationships between mathematics outcomes with IQ. Similar to adult findings, two studies found that higher FSIQ was related to better mathematics calculation skills (Berg et al., 2013) and with combined mathematics scores (Chapieski et al., 2011). The third study reported correlations between non-verbal intelligence with early numeracy in children with GGE. The final study found poor mathematics reasoning in children with epilepsy and below average IQ, but no differences were found in children with epilepsy and average IQ (Jones et al., 2010).

Four studies examined the relationship between working memory and mathematics skills. Of these, three studies found a relationship with mathematics outcomes. Working memory (measures of the phonological loop and central executive) was significantly correlated with early numeracy in children with GGE (Cheng et al., 2017), whereas attention and executive functioning were not. Persistence of seizures was related to poor working memory in focal epilepsies, which in turn predicted large deficits in mathematics calculation in children with epilepsy (Danguecan & Smith, 2017). This study also found a significant relationship between reduced processing speed and poorer mathematics skills. Finally, significant relationships between all components of working memory (phonological loop, central executive and visuo-spatial sketchpad) and processing speed with mathematics calculation outcomes (Reilly et al., 2014), however, only processing speed remained significant after controlling for FSIQ. In contrast, only one study found no relationship between mathematics calculation scores and working memory in children with mixed epilepsies (Ng & Hodges, 2020). Instead, that study found a relationship between processing speed, global ability, and attention with mathematics calculation in children with epilepsy.

#### Mathematics Anxiety

No included studies examined mathematics anxiety in adults or children with epilepsy.

### Publication Bias

No significant publication bias was detected with Egger’s regression test for mathematics calculation or the combined mathematics outcomes across studies in adults with epilepsy (*ps =* 0.74 − 0.877), and for early numeracy, mathematics calculation or mathematics reasoning across studies in children with epilepsy (*ps =* 0.088 − 0.19).

### Sensitivity Analysis

#### Adult Studies

For the combined mathematics outcome, adults with epilepsy, pooled across all subtypes, performed significantly worse than healthy adults in studies that were considered high selection quality (*k* = 8, *g*= -0.553, 95% CI -0.859, -0.247, *p* < .001), with significant large heterogeneity found for these studies (Q = 99.987, *df* = 11, *p* < .001, T^2^ = 0.249, *I*^*2*^ = 88.999, 95% prediction interval − 1.8324, 0.7264). For studies that were considered low selection quality, effect sizes were larger in magnitude on combined mathematics outcomes, compared to high quality studies (*k* = 12, *g*= -0.773, 95% CI -1.141, -0.405, *p* < .001), and significant large heterogeneity remained (Q = 244.657, *df* = 13, *p* < .001, T^2^ = 0.435, *I*^*2*^ = 94.686, 95% prediction interval − 2.3009, 0.7549).

#### Child Studies

For the combined mathematics outcome, children with epilepsy, pooled across all subtypes, performed significantly worse than typically developing children in studies that were considered high selection quality (*k* = 37, *g*= -0.580, 95% CI -0.716, -0.443, *p* < .001), with significant large heterogeneity found for these studies (Q = 205.699, *df* = 44, *p* < .001, T^2^ = 0.152, *I*^*2*^ = 78.61, 95% prediction interval − 1.3841, 0.2241). For studies that were considered low selection quality, effect sizes were larger in magnitude on combined mathematics outcomes, compared to high quality studies (*k* = 30, *g*= -0.609, 95% CI -0.822, -0.395, *p* < .001), and significant large heterogeneity remained (Q = 808.752, *df* = 34, *p* < .001, T^2^ = 0.382, *I*^*2*^ = 95.796, 95% prediction interval − 1.8946, 0.6766).

## Discussion

To our knowledge, this is the first meta-analysis examining specific early numeracy and secondary mathematics skills in adults and children with epilepsy. The primary aim of this review was to evaluate and quantify the gravity of deficits in early numeracy skills and secondary mathematics skills in adults and children with epilepsy and determine whether these skills are differentially impaired according to site of seizure focus. This meta-analysis revealed that both adults and children with epilepsy experienced significant impairments across a range of different mathematics outcomes.

For adults with epilepsy, this meta-analysis revealed moderate impairments in mathematics overall, with the greatest impairment found in mathematics reasoning, which was the most studied outcome in adults (*n* = 17). However, most studies used the Arithmetic subtest from the WAIS assessment battery, which involves oral delivery of mathematics questions which are timed. As a result, poor performance on this subtest may not only reflect difficulties with numerical skill and mathematics reasoning, but also poorer attention and working memory (Weiss et al., 2016; Karzmark, 2009). Poor performance can also be reflective of underlying learning difficulties and Attention Deficit Hyperactivity Disorder (ADHD; Hishinuma 1998). Only one study measured mathematics reasoning using a validated numeracy scale in adults with epilepsy and found that they significantly underperformed in mathematics reasoning compared to a healthy control sample of 1009 participants (Choi et al., 2011). Of interest, adults with epilepsy had significantly higher levels of education compared to the control group, suggesting that poor mathematics reasoning was not due to lower level of education and the result was not confounded by time limited mathematics reasoning assessment.

Deficits in early numeracy skills were found in adults with right and left TLE (Delazer et al., 2004), providing preliminary evidence of impaired early numeracy skills in TLE. Two studies evaluated mathematics calculation in adults with TLE and found moderate impairments, but this was not significant, perhaps due to the heterogeneity of epilepsy participants and method used to evaluate mathematics calculation. One study recruited adults with TLE with comorbid reading difficulties and found that adults with TLE had significantly impaired calculation performance despite having average or low IQ. This study found that adults without comorbid reading difficulties were not impaired in mathematics calculation (Breier et al., 2000). The second study by Delazer and colleagues (2004) used an experimental non-standardized assessment of arithmetic calculation and found that participants were impaired relative to controls. Moderate impairments in mathematics reasoning were revealed in TLE, and moderate-to-large deficits in GGE. No studies examined either early numeracy or calculation difficulties in other focal epilepsies and GGE. Given the small number of studies that have examined each mathematics outcome in adults with epilepsy, it is unclear whether mathematics difficulties are specific or pervasive across different types of epilepsy.

A greater number of studies examined mathematics outcomes in children with epilepsy. The greatest impairment found in children was in mathematics calculation (g = 0.762) followed by mathematics reasoning (g = 0.572) and early numeracy (g = 0.383). With respect to the site of epilepsy focus, no studies examined early numeracy skills in focal epilepsy. The only study that investigated early numeracy skills in GGE found a moderate impairment relative to healthy controls (Cheng et al., 2017). Mathematics calculation was evaluated in Extra-TLE/FLE and whilst large impairments were observed, this was not statistically significant, possibly due to the heterogenous nature of extra-TLE/FLE and the small sample sizes for each study. For mathematics reasoning, large deficits were revealed in FLE, and small-to-moderate impairments were observed in TLE and Extra-TLE/FLE. In contrast, GGE was the most negatively impacted, with moderate-to-large deficits in both mathematics calculation and mathematics reasoning, suggesting a possible global mathematics impairment across outcomes. Our findings are consistent with studies that found children with GGE have greater impairments in mathematics compared to children with focal epilepsies (e.g., Jackson et al., 2013; Rathouz et al., 2014).

The secondary aims of this review were to determine whether early numeracy and mathematics skills were related to demographic and epilepsy factors. Meta-regressions revealed that neither age at testing, age of onset, nor duration of epilepsy were associated with any of the mathematics outcomes for both adults and children with epilepsy. This result is contrary to studies that have found age of onset and duration of epilepsy having a deleterious impact on a range of neuropsychological outcomes (Hermann et al., 2002; Vendrame et al., 2009), but consistent with a longitudinal study that found mathematics deficits were present at the time of diagnosis in children with focal epilepsy and GGE, which persisted at 5 years follow up (Rathouz et al., 2014). The lack of association found between these epilepsy risk factors, and mathematics skills may also be due to the small number of studies included in the meta-regression, leading to low statistical power to detect an effect (Schmidt, 2017). Despite the lack of association between these variables and mathematics skills in epilepsy, other constitutional factors in epilepsy may contribute to mathematics difficulties.

The impact of seizure frequency was examined in children only, with mixed findings. Whilst some studies found that higher frequency of seizures were related to poorer mathematics outcomes (Adewuya et al., 2006; Bohac & Wodrich, 2013; Jones et al., 2010; Reilly et al., 2014; Smith et al., 2002), other studies found no relationship between seizure frequency and mathematics outcomes (Bailet & Turk, 2000; Huberty et al., 1992; Lopes et al., 2013; Ng & Hodges, 2020). The inconsistency in these findings may be related to the differences in how seizure frequency data was collected, defined, and analyzed across studies. For example, some studies categorized seizure frequency into a four-point scale (Smith et al., 2002), eight- or nine-point scales (Huberty et al., 1992; Ng & Hodges, 2020), or recorded the frequency of experienced seizures either weekly (Reilly et al., 2014), in the past month (Adewuya et al., 2006; Lopes et al., 2013), or past year (Jones et al., 2010), or simply compared the presence of seizures compared to no seizures (Bailet & Turk, 2000). Furthermore, some types of epilepsies can be well controlled with medication, resulting in lower frequency of seizures, whereas intractable or difficult to control seizures can lead to increased ASM use or require surgery, which may also result in significant cognitive impairments (Vingerhoets, 2006).

The relationship between ASM and mathematics was examined in adult and child studies. Higher ASM dosages, polytherapy, and some types of medications can be related to an increased risk of adverse cognitive effects, including a deleterious impact on working memory (Eddy et al., 2011; Park & Kwon, 2008), which in turn could impact mathematical skills. For adults with epilepsy, one study found that a higher number of ASMs were related to poorer mathematics outcomes (Traianou et al., 2019), another found no correlation between ASMs and mathematics (Abarrategui et al., 2018). Specifically, Traianou and colleagues (2019) included patients Extra-TLE/FLE who were on polytherapy and found that a higher number of ASMs was associated with poorer arithmetic calculations. Abarrategui and colleagues (2018) included patients with GGE on Valproate monotherapy and found no correlation between a valproate dose and mathematical skills. At a first glance, findings of these two studies suggest that polytherapy may be a risk factor for impaired mathematics skills. Nevertheless, participants in the study by Traianou and colleagues (2019) may have had complex epilepsy that was more difficult to control with medication. In children, those on valproate had worse (i) early numeracy skills, compared to children taking other ASM medications (Zhang et al., 2020), and (ii) mathematical skills compared to children taking carbamazepine (Bailet & Turk, 2000). Nonetheless, one study found that poorer arithmetic skills in children with idiopathic epilepsy treated with valproic acid at seizure onset, improved at subsequent follow ups, which raises a possibility that as children adjust to valproic acid, their mathematics performance improves (Bailet & Turk, 2000). Sulthiame, on the other hand, was related to a decline in mathematics achievement after six months of treatment despite achieving effective seizure control in a small study (n = 6) of children with BECTS (Wirrell et al., 2008). When comparing outcomes for those who do not take medication for their epilepsy, poorer mathematical outcomes were found in children who were taking ASMs (Berg et al., 2013). With respect to polypharmacy, only one study found that a greater number of ASMs were related to poorer mathematics outcomes (Reilly et al., 2014). In contrast, four studies found that the number of ASMs were unrelated to mathematics outcomes, and instead poor mathematics were related to seizure frequency (Danguecan & Smith, 2017; Smith et al., 2002), other cognitive skills (Ng & Hodges, 2020), or no significant relationship was found due to small sample sizes (Lopes et al., 2013). Taken together, whilst there is mixed evidence that polytherapy has a direct impact on mathematics skills in children with epilepsy, there is some evidence that the type of medication (e.g., Valproate and Sulthiame) may have a deleterious impact on mathematics skills.

Epilepsy surgery may lead to a reduction of seizures. Given that seizures negatively impact school attendance, this reduction of seizures may, in turn, increase school attendance and learning opportunities, and improve functional outcomes (Aguiar et al., 2007). No pediatric study found an improvement in mathematical skills from pre- to post-surgery. Instead, a decline in mathematics calculation scores was found irrespective of seizure status post-surgery (Puka et al., 2015). Nevertheless, the rate of decline differed; children who continued to experience seizures post-surgery had a greater decline compared to those who were seizure free. In other studies, no changes in mathematical skills were found in children who underwent focal temporal (Miranda & Smith, 2001), or temporal, frontal or extra temporal/frontal resections (Martin et al., 2016). In these two studies, participants were not impaired in mathematics prior to epilepsy surgery and had higher levels of IQ pre-surgery, compared to Puka and colleagues (2015). Only one adult study examined mathematical reasoning in participants who had adequate mathematical skills prior to temporal lobe surgery. One year post surgery an improvement was found following left, but not right, temporal resection (Davies et al., 1995).

The final aim of this review was to evaluate relations between early numeracy and mathematics outcomes with other factors: cognitive skills (e.g., working memory), or mathematics anxiety. With respect to cognitive skills, higher FSIQ was consistently found to be related to better mathematics skills in studies that included adults (Choi et al., 2011) and children with epilepsy (Berg et al., 2013; Chapieski et al., 2011; Jones et al., 2010). One study found that global cognitive ability was also related to mathematics outcomes in children with epilepsy (Ng & Hodges, 2020). Relationships with working memory were examined only in children but not adults with epilepsy. Two studies found greater working memory capacity to be related to better mathematical skills, including early numeracy skills in GGE (Cheng et al., 2017) and mathematics calculation in focal epilepsies (Danguecan & Smith, 2017). These findings are consistent with a meta-analysis that revealed early numeracy and calculation skills were associated with working memory capacity in typically developing children and adults (Peng et al., 2016). One study found that each component of working memory and processing speed were related to mathematics calculation, however, only processing speed remained significantly correlated with mathematics outcomes after controlling for FSIQ (Reilly et al., 2014). This study also included a high number of children with intellectual disability and reduced processing speed, which may better explain mathematics difficulties than working memory. Processing speed was also found to be related to mathematics in two other studies (Danguecan & Smith, 2017; Ng & Hodges 2020). Finally, Ng and Hodges (2020), found that attentional problems, rather than working memory, were related to mathematics outcomes in children with epilepsy. However, participants in this study had fewer difficulties with mathematics at assessment and a higher proportion of participants were diagnosed with ADHD compared to Reilly et al. (2014). Both Reilly et al. (2014) as well as Ng and Hodges (2020) also included mixed epilepsy samples (i.e. focal and generalized epilepsy), thus the relationship between working memory and mathematics in children with specific types of epilepsy is less clear. Given that working memory deficits were differentially vulnerable to the site and site of epilepsy focus (Poole et al., 2021), it may be hypothesized that children with GGE that experienced global deficits in working memory may have greater impairments in mathematics, compared to children with working memory components that were selectively impaired, such as those with unilateral TLE. Whilst this may be a plausible hypothesis, there are several other cognitive mechanisms that underpin mathematics difficulties, such as spatial skills (Bartelet et al., 2014) which was not investigated in studies included in this review.

No studies included in this review examined the impact of mathematics anxiety on mathematics outcomes in children with epilepsy. The lack of research in the impact of mathematics anxiety in epilepsy is surprising, given a recent meta-analysis has revealed that children and adolescents with epilepsy report greater levels of general clinical anxiety than controls (Scott et al., 2020). Furthermore, whilst it has been established that mathematics anxiety not only hinders mathematics achievement and learning (Barroso et al., 2021; Tomasetto et al., 2021) but also interferes with working memory (Ashcraft & Kirk, 2001), which is of particular interest in this clinical population.

### Limitations of the Literature and Current Review

Several methodological limitations of the current review need to be acknowledged. First, due to limited resources, only papers published in the English language were included in the review, thus papers published in other languages would have been missed. This review also included published peer-reviewed papers and excluded the grey literature as the latter could be of mixed quality. As a result, publication bias may be inflated in this review, but the included papers may be of higher quality. The quality analysis revealed that there may be potential for bias as few studies reported the non-response rate for epilepsy and control groups. There was also significant heterogeneity observed across studies, which may reflect the diverse range of epilepsy subtypes and syndromes included in this review. Sensitivity analyses revealed that studies with low selection quality resulted in larger effect sizes for both adult and child studies, indicating that bias may be present in those studies. Whilst studies that were identified as high selection quality had lower effect sizes, the findings remained significant. It should be acknowledged that despite studies being identified as high or low selection quality according to the NOS criteria, participants with epilepsy that have functional or academic concerns are more likely to be referred for neuropsychological assessment and included in research studies and may not be representative of the broader epilepsy population. Furthermore, significant heterogeneity remained for both high and low selection quality studies, which may reflect the diverse range of epilepsy subtypes and syndromes included in this review.

With respect to limitations of the literature, a large number of studies were excluded during the review process (n = 52) for not reporting mathematics outcomes separately to other academic outcomes (e.g., reading and writing) or did not report the arithmetic subtest score from an intellectual battery administered (e.g., from the WISC). While the arithmetic subtest score from the WISC is included in the working memory index score, according to the test publisher, arithmetic subtest assesses mathematics reasoning skills, as it requires of participants to answer a series of mental arithmetic questions and is also highly correlated with mathematics achievement (Weiss et al., 2016). As a result, the arithmetic subtest was included as a test of mathematics reasoning, when reported (or supplied via e-mail) separately from other WISC or WAIS scores. However, given that the arithmetic subtest requires speeded responses to orally delivered questions, performance on these measures may differ to mathematics reasoning outcomes in untimed paper and pencil format testing.

Several additional limitations of the child and adult literature are that few studies (i) examined early numeracy skills; (ii) reported mathematics outcomes separately for specific focal epilepsies, such as FLE; and (iii) examined the impact of epilepsy variables, cognitive skills, or mathematics anxiety on mathematics outcomes. Given that epilepsy variables often have deleterious impact of brain development and cognition, investigating the role of these variables on each component of mathematics could further inform clinicians on the increased risks to mathematics learning and performance so that targeted assessment and interventions can be provided.

Another limitation is that only a small number of studies examined mathematics outcomes in adults. This is an important shortcoming as difficulties with early numeracy and calculation skills can lead to health-related consequences, with adults with epilepsy experiencing greater difficulties accurately assessing risks related to treatment of their epilepsy (Choi et al., 2011). Furthermore, poor mathematics ability is known to be associated with unemployment and reduced income in adulthood in the general population (Parsons & Bynner, 1997; Rivera-Batiz, 1992). Given the real-world functional outcomes of poor mathematics ability – greater understanding of the impact of mathematics difficulties in adults with epilepsy is an important area to investigate further, so that practical assistance and supports can be provided to adults with epilepsy in order to engage successfully in the workforce or higher education, and also manage their epilepsy.

### Future Directions

This review has highlighted deficits in mathematics in both adults and children with epilepsy. Nevertheless, the mechanism that underpins these deficits requires further research. First, research into early numeracy skills of people with epilepsy is in its infancy and needs to be extended as poorly developed early numeracy skills may contribute to difficulties learning secondary mathematics skills (Mazzocco et al., 2011). Second, studies that examined relationship between working and mathematics in epilepsy were restricted to auditory working memory tasks, future studies should examine the role of the visuo-spatial sketchpad, which is also known to relate to mathematics outcomes (Mammarella et al., 2018). Third, it is important to further examine the impact of executive functions and processing speed deficits on mathematical skills in patients with epilepsy (Chan & Scalise, 2022; Cragg et al., 2017; Fuchs et al., 2006). Fourth, the role of various epilepsy variables in mathematical difficulties of people with epilepsy is under-researched. Detailing the mechanisms and factors that underpin mathematics difficulties in people with epilepsy may result in bespoke, comprehensive interventions for remediation of these difficulties.

Thorough neuropsychological assessment of mathematics difficulties can yield targeted supports and intervention to support people with epilepsy. For instance, in the general population, there are a broad range of possible interventions available. Difficulties with early numeracy skills have been shown to be remediated with number line training, which improved early numeracy skills and increased functional brain connectivity (Michels et al., 2018). Other interventions include targeted mathematics instruction, behavioral and psychological supports, non-invasive brain stimulation, and pharmacotherapy (Cohen Kadosh et al., 2013; Furlong et al., 2016). Yet no known interventions for mathematics difficulties have been trialed in epilepsy. If working memory deficits underpin mathematics difficulties, as indicated in one study (Danguecan & Smith, 2017) a range of working memory supports, and interventions may be explored. For instance, a computerized working memory training program, Cogmed has been used in children with epilepsy, which found improvements in working memory capacity (Kerr & Blackwell, 2015), with improvements maintained at three-month follow up (Fuentes & Kerr, 2017). However, one meta-analysis found no improvements in mathematics calculation after working memory training in the general population (Melby-Lervag & Hulme, 2013), thus further research is needed to determine whether improvements in working memory after training results in improvements in mathematics outcomes in people with epilepsy. Alternatively other supports may be indicated, such as classroom and teaching adjustments that reduce the load on working memory capacity, to better support mathematics learning and performance (Dehn et al., 2015). However, the effectiveness of these strategies in the general population is also mixed (Rowe et al., 2019). One study found improvement in mathematics after a working memory intervention in typically developing children (Colmar et al., 2020), suggesting that future research into working memory supports and whether they influence mathematics learning and performance is warranted.

Finally, future research is needed to investigate whether mathematics anxiety contributes to poor mathematics outcomes in epilepsy. This is an important area for further research given the clinical implication, as this would require clinicians to complete a separate assessment and provide concurrent psychological interventions for mathematics anxiety (Bicer et al., 2020), or provide appropriate and tailored intervention, such as a mathematics training program that fosters self-efficacy and accomplishment by adjusting the level of difficulty that corresponds to the child’s level of ability (Jansen et al., 2013). Another intervention program that focused on targeting early numeracy skills and working memory training, not only improved mathematics outcomes – but also remediated mathematics anxiety (Ng et al., 2022). This program may be of interest for further research, given the poor working memory comorbidity found in pediatric epilepsy (Poole et al., 2021).

## Conclusion

To our knowledge, this is the first fine-grained systematic review and meta-analysis examining specific mathematics outcomes in both adults and children with epilepsy. This review found that both adults and children with epilepsy have deficits in mathematics. Adults with epilepsy had significant impairments in mathematics reasoning, with only two studies reporting no significant deficits in mathematics calculation, and an insufficient number of studies examined early numeracy skills. According to site of seizure focus, adults with GGE experienced the greatest magnitude of impairment in mathematics reasoning, followed by TLE. For children with epilepsy, significant deficits were observed across all mathematics domains: early numeracy, mathematics calculation and mathematics reasoning, with the greatest magnitude observed for mathematics calculation – which is known to be strongly reliant on working memory capacity. According to site of seizure focus, children with GGE had significant deficits in both mathematics calculation and reasoning. Mathematics reasoning was significantly impaired in TLE, FLE and extra-TLE/FLE with the greatest magnitude of impairment observed for FLE. Mathematics calculation was not significantly impaired in extra-TLE/FLE, with an insufficient number of studies examining TLE or FLE. An insufficient number of studies also examined early numeracy skills across site of seizure focus.

It is important for clinicians and educators to recognize that people with epilepsy, especially those with GGE, are at an increased risk of mathematical difficulties, so that targeted screening and assessments can be conducted and appropriate interventions or supports put into place to ameliorate mathematical difficulties and their impact on people’s lives.

## Data Availability

The data that support the findings of this study are available from the corresponding author upon request. We would like to thank the researchers who were very generous in providing access to their data upon request.
